# Enhancing Automation and Interpretability of Vibrational Spectra Predictions for Water Clusters from Diffusion Monte Carlo

**DOI:** 10.1021/acs.jpca.5c03743

**Published:** 2025-09-16

**Authors:** Sijing Zhu, Lindsey R. Madison

**Affiliations:** Department of Chemistry, 8439 Colby College, Waterville, Maine 04901, United States

## Abstract

A method for predicting vibrational spectra of water clusters ${\left(H_{2}O\right)}_{n=2,3,4,6}$ from wave functions sampled by diffusion Monte Carlo (DMC) is developed to enhance automation, generalizability, and interpretation. This method builds on the established ground-state probability amplitude (GSPA) approach to vibrational spectra predictions and is applied to neutral water clusters, systems defined by high dimensionality and having significant nuclear quantum effects. We develop a chemically informed singular value decomposition (SVD) approach to automate the selection of internal coordinates for vibrational analysis, along with an optimization-based reverse mapping method to visualize vibrational motions in Cartesian space. Both developments are generalized to handle water clusters of varying sizes. The framework is assessed on the q-SPC/Fw potential energy surface, and we find that the chemically informed SVD yields accurate and basis-invariant spectroscopic predictions across all clusters studied, fully addressing the bias observed toward intermolecular overrepresentation of the standard SVD approach. Additionally, we systematically benchmark DMC sampling and descendant weighting convergence to ensure the reliability of the ground-state probability amplitude inputs used in the vibrational analysis. Together, these developments establish an automated and interpretable framework for vibrational spectra prediction from DMC, with potential applicability to a wide range of molecular systems beyond water clusters.

## Graphical abstract

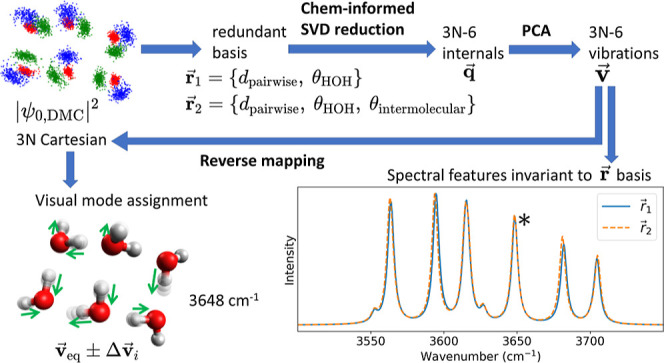

## Introduction

Water, ubiquitous and vital, has properties spanning scales from molecular to bulk that are fundamentally influenced by nuclear quantum effects (NQE). NQE arise from the delocalized nature of atomic nuclei and are inherently reflected in the spectroscopy of molecular vibrations. Neglecting NQE in molecular simulations leads to inaccurate predictions of thermodynamic properties such as thermal expansion, density, and heat capacity,^1–5^ vibrational properties such as proton transfer dynamics and hydrogen bond rearrangement,^3,6^ and electronic properties such as band gap.^7^ NQE are reflected in vibrational spectra of water clusters and theoretical predictions of the vibrational motions of these systems can provide insights into how molecular interactions give rise to macroscopic properties.^8^

The vibrational ground state is obtained from solving the Schrödinger equation. While the full vibrational wave function for water clusters cannot be determined analytically, Diffusion Monte Carlo (DMC)^9^ offers a numerical alternative by sampling the wave function using a random walk process. DMC has been shown to accurately capture anharmonicity, large-amplitude motions, and mode coupling of aqueous systems.^10–12^ Previous DMC studies^13–19^ of NQE of neutral water have primarily focused on clusters ${\left(H_{2}O\right)}_{n,2\leq n\leq 6}$ because they represent transient structures commonly observed in bulk water^20–23^ and also have a reasonable computational cost. These studies have developed methods to improve the efficiency of DMC calculations through importance sampling,^11,13,24,25^ examined NQE within and across conformations,^14,26^ and computed ground-state vibrational properties including rotational moments of inertia,^14^ oxygen–oxygen pair distance distributions,^16^ and binding energies.^14^ The DMC wave function in Cartesian coordinates, combined with descendant weighting,^27^ yields the ground-state probability amplitude (GSPA), which has been successfully applied to predict vibrational spectra for charged water clusters, such as protonated water tetramer^12^ and the deprotonated water dimer^28^ including up to two quanta of excitation. The GSPA approach projects the wave funciton sampling, known as walkers, onto a set of selected 3*N* – 6 internal coordinates, and uses a principal component analysis (PCA) to extract the principal axes of motions, which after mass weighting, resembles the orthogonal vibrational modes. The use of mass-weighted PCA to extract vibrational coordinates is conceptually similar to principal mode analysis (PMA), originally developed by Wheeler et al.,^29^ furthered by Schmitz and Tavan,^30^ and later applied to systems such as polycyclic aromatic hydrocarbon molecules.^31^ The key distinction is that GSPA relies on wave function sampling from DMC, whereas PMA is based on classically sampled distributions, such as molecular dynamics trajectories. In PMA, the PCA eigenvalues are directly used to estimate vibrational frequencies within an effective harmonic treatment. In contrast, GSPA introduces vibrational excitations by explicitly inserting nodes into the ground-state amplitude using excitation polynomials along the extracted vibrational coordinates. Frequencies and intensities are then computed from matrix elements using Monte Carlo integration.^28^ Although the accuracy of GSPA has been benchmarked in prior work,^28^ we further demonstrate that, for a one-dimensional Morse oscillator, GSPA yields more accurate frequencies than an effective harmonic treatment, particularly in regimes of strong anharmonicity (Table S1).

While prior applications of GSPA have utilized manually selected internal coordinates,^12,28,32^ different internal coordinate selection schemes can impact the quality of GSPA-derived vibrational spectra. Additionally, the selected coordinates must be complete and nonredundant while maintaining chemical interpretability, which is crucial to enable mode assignment. These requirements become increasingly difficult to satisfy for larger, more flexible water clusters involving delocalized motions, where the choice of internal coordinates depends sensitively on the specific geometry.

In this work, an approach to streamline internal coordinate selection for the GSPA method is developed and applied. We compared two redundancy reduction schemes that algorithmically select 3*N* – 6 linear combinations from a large set of redundant internal coordinates comprising bond lengths, bond angles, and all intermolecular atomic distances. One scheme applies a standard singular value decomposition (SVD),^33^ while the other is a chemically informed redundancy reduction method newly developed in this work, in which bond lengths and bond angles in the initial basis are explicitly retained, and the remaining coordinates are selected via SVD as orthogonal intermolecular descriptors.

Regardless of the internal coordinate selection technique used, whether manual or automated redundancy reduction, the vibrational modes extracted by GSPA can be challenging to interpret as they are linear combinations of the initial coordinate set. To enhance physical interpretability, we introduce an optimization-based reverse mapping algorithm that reconstructs a Cartesian geometry best approximating a given set of vibrational coordinates. This numerical procedure enables direct visualization of motions along vibrational coordinates in Cartesian space and streamlines the mode assignment process.

We systematically benchmark the spectroscopic predictions of GSPA methods using both standard and chemically informed redundancy reduction schemes across the water dimer, trimer, tetramer, and hexamer to assess the generalizability and consistency of the predictions with respect to the completeness of the redundant basis. Furthermore, to ensure the reliability of the input wave function and probability amplitude data for GSPA, we systematically evaluate the convergence behavior of DMC simulations with and without importance sampling, under both continuous and discrete weighting schemes, as well as the convergence of various vibrational properties in descendant weighting simulations. To enable efficient benchmarking, we selected the relatively simple q-SPC/Fw potential energy surface (PES),^2,34^ which uses harmonic intramolecular potentials and Coulombic and Lennard-Jones intermolecular interactions. Despite its simplicity, q-SPC/Fw nonetheless introduces the effects of coupled inter- and intramolecular degrees of freedom, making it a suitable test case for assessing the performance of the generalized GSPA method. In addition, the explicit parametrization of force constants enables the construction of an analytical guiding function for DMC simulations.

## Methods

### Overview of Diffusion Monte Carlo (DMC)

Diffusion Monte Carlo (DMC)^9^ projects the wave function onto the ground state by propagating the time-dependent nuclear Schrödinger equation in imaginary time, τ = *it*/ℏ. The technique applied to determine the nuclear wave function has been well described^9–11,35^ and a brief overview is provided here. For an *N*-atom system with nuclear coordinates **R**, the wave function Ψ­(**R**, τ) evolves according to

1 $$\frac{\partial \Psi \left(R,\tau \right)}{\partial \tau }=\sum_{i=1}^{N}\frac{\hbar^{2}}{2m_{i}}\nabla_{i}^{2}\Psi \left(R,\tau \right)-\left[V\left(R\right)-V_{ref}\right]\Psi \left(R,\tau \right)$$

where *m*_*i*_ is the mass of atom *i*, and *V*_ref_ is a dynamically adjusted simulation variable known as the reference energy which approximates the zero point energy (ZPE). At large τ, the excited-state components decay exponentially, and the wave function converges to the ground state ψ_0_(**R**).^10,35^

This imaginary-time Schrödinger equation is mathematically equivalent to a diffusion–reaction equation that governs the evolution of a particle density *C*(**R**, *t*) in a 3*N*-dimensional configuration space

2 $$\frac{\partial C\left(R,t\right)}{\partial t}=\sum_{i=1}^{N}D_{i}\nabla_{i}^{2}C\left(R,t\right)-k\left(R\right)C\left(R,t\right)$$

where the diffusion constant of atom *i* is *D*_*i*_ = ℏ^2^/(2*m*_*i*_), and the spatially dependent first-order rate term is *k*(**R**) = *V*(**R**) – *V*_ref_. This correspondence enables DMC to simulate imaginary-time wave function propagation of a system using an ensemble of fictitious particles, termed walkers, evolved under a finite time step Δτ.^9^ Each walker represents a configuration of the system. Within each walker, each atom diffuses independently to reflect the additive structure of the kinetic energy operator. The first order term is implemented as a growth or decay process known as branching^9^ and it acts on the walker as a whole, governed by the total potential energy of the walker configuration *V*(**R**) relative to the reference energy. As the number of iterations increases, the walker density converges to the ground-state wave function ψ_0_(**R**).^9^ This original formulation is referred to as discrete weighting DMC. An alternative is continuous-weighting DMC which maintains a fixed walker population with each walker’s weight *w*_*j*_ (initialized as one) undergoing exponential growth or decay.^11^ To prevent the total weight from becoming dominated by a few high-weight walkers, a resampling procedure is applied at each iteration: walkers with weights below a fixed threshold are removed, and an equal number of highest-weight walkers are duplicated, with their weights halved to conserve total weight. We use a threshold of 0.01 for the resampling step, consistent with the approach of Lee et al.^17^

To overcome the loss of sampling efficiency which has been shown to represent a significant challenge when modeling water clusters,^16^ the importance sampling approach developed by Mentch and Anderson^36^ was used. This method employs a guiding function, ψ_guide_, which, in addition to diffusion, introduces a drift motion that biases the walker distribution toward regions of configuration space where the ground-state amplitude is expected to be significant. Reynolds et al.^37^ later incorporated a Metropolis algorithm to probabilistically accept the combined drift and diffusion step, ensuring microscopic reversibility. A series of studies on water clusters from dimer to hexamer^13,17,38^ have guided the sampling of the monomer intramolecular bond stretching and bending using harmonic oscillator functions fitted to one-dimensional cuts of the MB-pol potential. This guiding function was shown to accurately approximate the wave function along intramolecular degrees of freedom and thus reduce the number of walkers required for convergence by up to an order of magnitude.

Diffusion Monte Carlo requires a specified potential energy surface (PES), *V*(**R**), to evaluate the wave function. Previous studies have employed the MB-pol PES^14,15,17^ and q-TIP4P/F.^16^ In this study, we use the q-SPC/Fw PES developed by Paesani et al.^2^ refined from the SPC/E PES by Wu et al.^34^ for which the analytical form and low computational cost make it well suited for efficiently benchmarking our vibrational analysis framework. The q-SPC/Fw PES consists of an intramolecular harmonic oscillator potential that sums bond stretching and angle bending terms within each monomer, explicitly parametrized by the force constants *k*_OH_ and *k*_A_, a Lennard-Jones potential for pairwise oxygen–oxygen interactions, and a Coulombic interaction for all intermolecular atom pairs. The parameters are provided in ref 2.

The explicit parametrization of the q-SPC/Fw PES using force constants *k*_OH_ and *k*_A_ enables the construction of an analytical guiding function that describes the three decoupled intramolecular motions as a product over all water monomers

$$\psi_{guide}=\overset{N_{mol}}{\underset{k=1}{\prod }}\exp\left\{-\frac{1}{2\hbar }\left[\begin{array}{l} \sqrt{k_{OH}\;\mu_{OH}}{\left(l_{OH1,k}-l_{eq}\right)}^{2}+\\ \sqrt{k_{OH}\;\mu_{OH}}{\left(l_{OH2,k}-l_{eq}\right)}^{2}+\\ \sqrt{k_{A}\;\mu_{A}}{\left(\theta_{k}-\theta_{eq}\right)}^{2} \end{array}\right]\right\}$$ 3

where *l*_OH1,*k*_ and *l*_OH2,*k*_ are the two OH bond lengths and θ_*k*_ is the HOH angle of monomer *k*. The reduced masses are computed as^39^

4 $$\mu_{OH}=\frac{m_{O}\cdot m_{H}}{m_{O}+m_{H}},,\mu_{A}=\frac{l_{eq}^{2}}{2}{\left(\frac{1}{m_{H}}+\frac{1-\cos\left(\theta_{eq}\right)}{m_{O}}\right)}^{-1}$$

Following DMC sampling, descendant weighting^27^ (DW) can be used to obtain an additional weight for each walker that reflects its contribution to the ground-state probability amplitude. In discrete weighting DMC, the descendant weight *W*_*j*_^DW^ for walker *j* is proportional to the number of its descendants after an additional imaginary time τ_DW_. In continuous-weighting DMC, the descendant weight is computed as^25^

5 $$W_{j}^{DW}=\frac{w_{j}\left(\tau +\tau_{DW}\right)}{w_{j}\left(\tau \right)}$$

where *w*_*j*_ is the continuous walker weight. Expectation values of any scalar, vector, or tensor observables Ô are computed as DW-weighted averages over walker configurations **R**_*j*_ ^25^

6 $$\left\langle O\right\rangle =\frac{\sum_{j}W_{j}^{DW}w_{j}Ô\left(R_{j}\right)}{\sum_{j}W_{j}^{DW}w_{j}}$$

For discrete weighting DMC, this expression applies with uniform weights *w*_*j*_ = 1.

Accurate sampling of the vibrational probability amplitude requires a sufficiently large τ_DW_. However, increasing τ_DW_ also leads to a more uneven distribution of descendant weights, which amplifies statistical noise.^10^ A prior benchmark study^11^ reported that τ_DW_ = 250 provides a reasonable balance between accuracy and precision. However, this recommendation was likely based primarily on high-frequency local stretching modes and has not been shown to generalize to more delocalized motions. Several studies^13,14^ have employed τ_DW_ values between 100 and 300 to compute isomer fractions, but without convergence validation. Given that vibrational observables may converge at different rates, we systematically benchmark descendant-weighting convergence across a range of vibrational properties.

### Investigating DMC Sampling Behavior on q-SPC/Fw PES

DMC sampling is known to be sensitive to the initial placement of walkers.^14,15,17,18^ Establishing unbiased initial conditions require a thorough exploration of the PES landscape. To this end, we combine Metropolis Monte Carlo with energy minimization to explore the various energy minima (isomers) on the q-SPC/Fw PES, using the approach of Mallory and Mandelshtam.^14^ Broyden–Fletcher–Goldfarb–Shanno (BFGS) optimization^40–43^ is used for its rapid convergence. Energy minimization enables classification of DMC walkers into characteristic isomers and has been used to compute the distribution of ground-state probabilities across isomers, referred to as the isomer fractions.^14^ To further distinguish between energetically equivalent conformations that differ only by atom relabeling, Eckart alignment^44^ is applied to compare each minimized walker to permutational variants of its assigned isomer. A numerically zero mass-weighted root-mean-square deviation (RMSD) indicates that the walker belongs to that specific permutation of the isomer. Permutation detection in DMC provides a means to identify energetically indistinguishable label exchanges that could otherwise distort vibrational calculations. To further characterize the potential energy landscape, we map the connectivity and energy barriers between isomers using the climbing-image nudged elastic band (CI-NEB) method,^45^ with image-dependent pair-potential path initialization.^46^ In addition to isomerizations, we use NEB to quantify the energetic cost of atomic label permutations (see Table S2).

### Overview of Ground State Probability Amplitude (GSPA) Approach to Spectroscopic Predictions

After obtaining the DMC wave function and associated probability amplitude, the vibrational spectra can be predicted following the Ground-State Probability Amplitude (GSPA) approach developed by McCoy and co-workers.^12,28,32^ GSPA extracts normal modes based on a ground-state wave function sampling. By directly exciting the ground-state wave function along each normal mode using Hermite polynomials, GSPA explicitly incorporates anharmonicity of the ground state and gives more accurate spectral predictions compared to effective harmonic treatment alone (Table S1). The approach is shown in eq 7 which summarizes the relationships between the coordinate representations and the transformations. In brief, the GSPA wave function, initially represented in Cartesian coordinates *x⃗*, is projected onto a manually selected set of internal coordinates *q⃗*, via a nonlinear transformation g(x⃗) . The internal coordinates are then transformed into a set of vibrational coordinates *v⃗* via the diagonalization of the covariance matrix of the internal coordinates, also known as the second moments matrix. The vibrational coordinates are then transformed into mass-weighted vibrational coordinates, ${\mathrm{v⃗}}_{MW}$ , allowing for energy evaluations.

7 $$\mathrm{x⃗}\overset{g\left(\mathrm{x⃗}\right)}{\to }\mathrm{q⃗}\overset{P^{T}}{\to }\mathrm{v⃗}\overset{{\left\langle G\right\rangle }^{-1/2}}{\to }{\mathrm{v⃗}}_{MW}$$

The transformation matrix **P**, whose transpose **P** ^*T*^ maps *q⃗* to *v⃗*, consists of the eigenvectors of the mass-weighted covariance matrix

8 $$P=diag\left(\langle {G\rangle }^{-1/2}C\langle {G\rangle }^{-1/2}\right)$$

where **C** is the covariance matrix, of the internal coordinates

9 $$C_{ij}=\left\langle \left(q_{i}-\left\langle q_{i}\right\rangle \right)\left(q_{j}-\left\langle q_{j}\right\rangle \right)\right\rangle$$

and ⟨**G**⟩ is the ensemble-averaged Wilson **G** matrix (3*N* – 6 × 3*N* – 6),^39^ which defines the global kinetic energy metric across the ground-state probability amplitude

10 $$\langle {G\rangle }_{ij}=\left\langle \sum_{k=1}^{3N}\frac{\partial q_{i}}{\partial x_{k}}\frac{1}{m_{k}}\frac{\partial q_{j}}{\partial x_{k}}\right\rangle$$

After mass-weighting the vibrational coordinates via inverse fractional power of **G**, $v_{MW,l}={\left\langle G\right\rangle }^{-1/2}{\mathrm{v⃗}}_{l}$ , relevant quantities for spectral predictions can be calculated for each mode *l*. The fundamental frequency ν_*l*_ for vibrational mode *l* is computed from the potential and kinetic energy gaps between the first excited state and the ground state

$${h\nu }_{1\to 0}=\left({\left\langle V\right\rangle }_{1,l}-{\left\langle V\right\rangle }_{0}\right)+\left({\left\langle T\right\rangle }_{1,l}-{\left\langle T\right\rangle }_{0}\right)$$ 11

A Hermite polynomial along each ${\mathrm{v⃗}}_{MW,l}$ is multiplied onto the ground-state wave function to excite each vibrational mode independently

12 $$\psi_{1,l}=f_{1,l}\psi_{0}=\left(v_{MW,l}-\left\langle v_{MW,l}\right\rangle \right)\psi_{0}$$

The expectation value of the ground-state potential energy follows eq 6, while the excited potential energy along mode *l* is computed as

13 $${\left\langle \mathrm{V̂}\right\rangle }_{1,l}=\frac{\left\langle Vf_{1,l}^{2}\right\rangle }{\left\langle f_{1,l}^{2}\right\rangle }=\frac{\sum_{j}^{N_{w}}f_{1,l}^{2}\left(v_{MW,l}\right)V\left(R_{j}\right)W_{j}^{DW}w_{j}}{\sum_{j}^{N_{w}}f_{1,l}^{2}\left(v_{MW,l}\right)W_{j}^{DW}w_{j}}$$

The kinetic portion of the excitation energy is related to the variance of the GSPA along *v*_MW,*l*_

14 $${\left\langle T\right\rangle }_{1,l}-{\left\langle T\right\rangle }_{0,l}\approx \frac{\hbar^{2}}{2}\frac{\left\langle v_{MW,l}^{2}\right\rangle }{\left\langle v_{MW,l}^{4}\right\rangle -{\left\langle v_{MW,l}^{2}\right\rangle }^{2}}$$

A more detailed discussion of the theoretical foundation of eqs 13 and 14 can be found in the Supporting Information.

The transition frequency is calculated The IR absorbance intensity of a vibrational transition is related to the excitation energy and the transition^47^

$$\begin{array}{ll} I_{l}\propto \nu_{1\to 0,l}{\left|\left\langle 0\left|\mathrm{\mu ⃗}\right|1_{l}\right\rangle \right|}^{2} & =\nu_{1\to 0,l}\frac{{\left|\left\langle \mathrm{\mu ⃗}f_{1,l}\right\rangle \right|}^{2}}{\sqrt{\left\langle f_{1,l}^{2}\right\rangle }\sqrt{\langle 0|0\rangle }}\\ & =\nu_{1\to 0,l}\frac{\overset{N_{w}}{\underset{j}{\sum }}f_{1,l}^{2}\left(v_{MW,l}\right)\mathrm{\mu ⃗}\left(R_{j}\right)W_{j}^{DW}w_{j}}{\sqrt{\overset{N_{w}}{\underset{j}{\sum }}f_{1,l}^{2}\left(v_{MW,l}\right)W_{j}^{DW}w_{j}}\sqrt{\overset{N_{w}}{\underset{j}{\sum }}W_{j}^{DW}w_{j}}} \end{array}$$ 15

where μ⃗ is the dipole moment vector that are computed from the partial charges parametrized by the q-SPC/Fw PES.

### Chemically Informed Automated Coordinate Selection

To address the challenges of manually selecting 3*N* – 6 internal coordinates, a fully automated approach is introduced and outlined in eq 16

16 $$\mathrm{x⃗}\overset{f\left(\mathrm{x⃗}\right)}{\to }\mathrm{r⃗}\overset{U^{T}}{\to }\mathrm{q⃗}\overset{P^{T}}{\to }\mathrm{v⃗}\overset{{\left\langle G\right\rangle }^{-1/2}}{\to }{\mathrm{v⃗}}_{MW}$$

A large set of *M* redundant internal coordinates *r⃗* is first computed from Cartesian coordinates. Different choices of redundant descriptors form different initial bases for coordinate selection and vibrational representation. In this study, we consider two such bases, ${\mathrm{r⃗}}_{N^{2}}$ and ${\mathrm{r⃗}}_{N^{3}}$ :

-
${\mathrm{r⃗}}_{N^{2}}$ : intramolecular OH bond lengths and HOH angles, along with all pairwise intermolecular atomic distances. This basis grows quadratically with system size.
-
${\mathrm{r⃗}}_{N^{3}}$ : defined as ${\mathrm{r⃗}}_{N^{2}}$ plus all intermolecular angles, yielding cubic scaling with system size.

To identify the 3*N* – 6 most physically meaningful and linearly independent coordinates from *r⃗*, we begin with the Wilson **B** matrix,^39^ which is a Jacobian matrix that maps infinitesimal Cartesian displacements δ*x⃗* to internal coordinate displacements δ*r⃗*

17 $$\delta \mathrm{r⃗}=B\delta \mathrm{x⃗},,B_{i,j}=\frac{\partial r_{i}}{\partial x_{j}}$$

To account for global geometric fluctuations, we compute ⟨**B**⟩, the expectation value of **B** matrix across the walker ensemble. This introduces no additional cost, as the **B** matrices for each walker are reused in constructing the ensemble average ⟨**G**⟩ (eq 18). This calculation of ⟨**G**⟩ is fully vectorized and thus more efficient compared to element-wise computation (eq 10).

18 $$\left\langle G\right\rangle =\left\langle U^{T}B\Lambda B^{T}U\right\rangle =U^{T}\left\langle B\Lambda B^{T}\right\rangle U$$

where **Λ** is a diagonal matrix of inverse atomic masses.

Singular value decomposition (SVD) is then applied to ⟨**B**⟩, yielding three matrices

19 $$\left\langle B\right\rangle =U^{\prime }\Sigma V^{T}$$

where the columns of **U**′ form an *M* × *M* matrix and are orthonormal linear combinations of the redundant internal coordinates, with their relative significance reflected in the corresponding singular values that are the diagonal elements of **Σ**. The first 3*N* – 6 columns of **U**′, associated with the largest singular values, are retained to define the coordinate reduction matrix **U** in eq 16:

As cluster size increases, the number of intermolecular coordinates in the redundant basis grows faster than that of intramolecular ones, biasing coordinate selection toward intermolecular motions and underrepresenting OH stretching and HOH bending modes.

To mitigate this imbalance, we developed a chemically informed SVD approach. The *K* intramolecular modes—two OH bond lengths and one HOH angle per water monomer—are fixed as the first *K* components of *q⃗* (with *K* = *N* for all water clusters studied). The remaining 3*N* – 6 – *K* intermolecular directions are selected to be orthogonal to the fixed intramolecular subspace, thereby ensuring nonredundancy and completeness. These directions are identified through the following procedure:

1. Construct a basis for the fixed subspace. Let ⟨**B**⟩_fixed_ (*K* × 3*N*) denote the rows of ⟨**B**⟩ corresponding to the fixed intramolecular coordinates. A QR decomposition of its transpose yields

$${\left\langle B\right\rangle }_{fixed}^{T}=Q_{f}R$$ 20

where $Q_{f}$ (3*N* × *K*) spans the fixed intramolecular subspace.

2. Apply orthogonal projection. Let ${\left\langle B\right\rangle }_{free}\left(M-K\right)\times 3N$ denote the rows of ⟨**B**⟩ corresponding to all unfixed (intermolecular) coordinates. These rows are projected into the orthogonal complement of the fixed subspace using the projection matrix ${P_{⊥}=I_{3N}-Q}_{f}Q_{f}^{T}$ , yielding

21 $$\langle {B\rangle }_{proj}={\left(P_{⊥}\langle {B\rangle }_{free}^{T}\right)}^{T}$$

3. Apply SVD to the projected matrix.

22 $${\left\langle B\right\rangle }_{proj}=U_{free}^{\prime }\Sigma_{free}V^{T}$$

The leading 3*N* – 6 – *K* columns of **U**_free_^′^, forming an *M* – *K* × 3*N* – 6 – *K* matrix, are retained to form **U**_free_, which selects intermolecular directions orthogonal to the fixed intramolecular motions.

4. Construct the final transformation matrix. The full transformation matrix **U** (*M* × 3*N* – 6), whose transpose maps *r⃗* to *q⃗* as defined in eq 16, is assembled as

23 $$U=\left[\begin{array}{ll} I_{K} & 0\\ 0 & U_{free} \end{array}\right]$$

where **I**_*K*_ preserves the fixed intramolecular coordinates and **U**_free_ selects the remaining orthogonal intermolecular ones. They preserve the orthonormality of the coordinate reduction, as in the standard SVD-based formulation.

With the internal coordinate basis defined and redundancy reduced, we have now established a general forward mapping from Cartesian coordinates to both unweighted and mass-weighted vibrational coordinates

24a $$\mathrm{v⃗}\left(\mathrm{x⃗}\right)=P^{T}U^{T}f\left(\mathrm{x⃗}\right)$$

24b $${\mathrm{v⃗}}_{MW}\left(\mathrm{x⃗}\right)={\left\langle G\right\rangle }^{-1/2}P^{T}U^{T}f\left(\mathrm{x⃗}\right)$$

### Optimization-Based Reverse Mapping for Visual Mode Assignment

To address the abstraction of vibrational mode representations, we introduce an optimization-based reverse-mapping framework that numerically reconstructs Cartesian geometries from a series of displacements in mass-weighted vibrational space, enabling visual mode assignment. We frame the reverse mapping as an optimization problem: starting from an initial guess ${\mathrm{x⃗}}_{0}$ , we seek the Cartesian geometry whose projected mass-weighted vibrational coordinates best match the target. The objective function quantifies the deviation between the target and the projected mass-weighted vibrational coordinates

25 $$err\left(\mathrm{x⃗}\right)={∥{\mathrm{v⃗}}_{MW}\left(\mathrm{x⃗}\right)-{\mathrm{v⃗}}_{MW,target}∥}^{2}$$

This error is defined as the sum of square error and iteratively minimized using the BFGS algorithm until falling below a threshold (<10^–8^), yielding a Cartesian geometry that reproduces the target vibrational coordinates.

Given a target ${\mathrm{v⃗}}_{MW,target}$ and an initial condition ${\mathrm{x⃗}}_{0}$ , we denote the solution obtained by the algorithm as

26 $${\mathrm{x⃗}}_{sol}={\mathrm{v⃗}}_{MW}^{-1}\left({\mathrm{v⃗}}_{MW,target},{\mathrm{x⃗}}_{0}\right)$$

To visualize vibrational modes, we first solve for the equilibrium geometry ${\mathrm{x⃗}}_{eq}$ corresponding to zero displacements in mass-weighted vibrational space, using the global minimum structure ${\mathrm{x⃗}}_{GM}$ as the initial guess

27 $${\mathrm{x⃗}}_{eq}={\mathrm{v⃗}}_{MW}^{-1}\left(0⃗,{\mathrm{x⃗}}_{GM}\right)$$

For each vibrational mode direction ${\mathrm{v⃗}}_{MW,i}$ , we then perturb the mass-weighted vibrational coordinates incrementally in the positive and negative directions

28 $${\mathrm{v⃗}}_{MW,target,k}^{\left(+\right)}=+k\delta {\mathrm{v⃗}}_{MW,i}$$

29 $${\mathrm{v⃗}}_{MW,target,k}^{\left(-\right)}=-k\delta {\mathrm{v⃗}}_{MW,i}$$

Each set of perturbed mass-weighted vibrational coordinates is reverse mapped to a Cartesian geometry using the previous structure as the initial guess

30 $${\mathrm{x⃗}}_{k}^{\left(+\right)}={\mathrm{v⃗}}_{MW}^{-1}\left({\mathrm{v⃗}}_{MW,target,k}^{\left(+\right)},{\mathrm{x⃗}}_{k-1}^{\left(+\right)}\right)$$

31 $${\mathrm{x⃗}}_{k}^{\left(-\right)}={\mathrm{v⃗}}_{MW}^{-1}\left({\mathrm{v⃗}}_{MW,target,k}^{\left(-\right)},{\mathrm{x⃗}}_{k-1}^{\left(-\right)}\right)$$

These sequences of Cartesian displacements can be animated to visualize the vibrational motions and assign their characters.

### Simulation Conditions

DMC simulations were performed with a total propagation time of τ = 50, 000 (with an exception of data in Figure S6), a time step of Δτ = 1, and an equilibration period of τ_eq_ = 30,000; during the initial τ = 50 steps of τ_eq_, only diffusion was applied (branching was disabled) to reduce early energetic bias and promote broader sampling of the PES; after equilibration, a total of 20 wave function snapshots were collected at even intervals over the final τ_collection_ = 20,000, and ZPEs were computed by averaging *V*_ref_ values from each iteration during this collection period.

All wave function properties, such as the wave function distribution along internal coordinates or among isomers, are computed from $\psi_{0}=\psi_{DMC}/\psi_{guide}$ , where the contribution of each walker to the associated property is weighted by 1/ψ_guide_.

Multiple independent DMC repetitions were performed. When treated separately (denoted *N*_DMC_), they were used to estimate standard deviations; when concatenated into a single set of wave function data, denoted *N*_DMC_^c^, they reduced statistical noise. Descendant weighting simulations were applied to each individual wave function snapshot. For each snapshot, descendant weights were averaged over *N*_DW_ repetitions to mitigate the disproportionate influence of minority walkers with large weights. All calculations involving expectation values in this study used *N*_DW_ = 3. Vibrational properties were benchmarked using τ_DW_ = 250 – 4000.

All coordinate-based analyses in the GSPA method employed Eckart alignment^44^ of the walkers to the global minimum (GM) structure, minimizing the rotation-vibration coupling. Unless otherwise noted, all DMC wave functions were initialized using a consistent atom-labeling permutation to facilitate subsequent vibrational analyses. Spectral predictions employed continuous-weighting guided DMC with *N*_DMC_^c^ = 5, τ_DW_ = 1, 000, and *N*_DW_ = 3, using *N*_*w*_ = 5,000 (monomer), 10,000 (dimer), 50,000 (trimer), and 100,000 (tetramer and hexamer).

## Results and Discussion

### Convergence Behavior of DMC on q-SPC/Fw PES

To start, the convergence of the ground-state wave functions of varying water cluster sizes with respect to ZPE and isomer populations is assessed. Figure 1 shows the convergence of ZPE with respect to the ensemble size as a function of 1/*N*_w_, a well-established metric for assessing convergence in DMC simulations,^13,16,17^ across four variations of DMC implementation. Continuous and discrete weighting DMC exhibit nearly identical convergence behavior across all cluster sizes. Guided DMC reduces the walker population needed for convergence, particularly in larger systems, thereby improving the efficiency of both wave function sampling and subsequent GSPA spectral predictions. The *N*_w_ employed in the spectroscopic calculations, as specified in the Simulation Conditions section, were selected in accordance with the convergence behavior demonstrated. For all clusters studied, the ZPEs from guided and unguided DMC converge, on average, within 0.01 kcal/mol of each other and are statistically indistinguishable (see Table S3). In addition, wave functions generated by unguided and guided DMC, projected along various internal coordinates, show complete agreement across all clusters studied (see Figure S1-4). Both methods generate wave function projections with qualitatively reasonable anharmonic shifts and broadening. Thus, the use of an analytical intramolecular guiding function does not lead to significant bias. This is in agreement with the results of Lee et al.,^13^ who used numerically fitted harmonic functions along one-dimensional PES cuts. Additionally, continuous-weighting guided DMC yields significantly less statistical noise than discrete weighting (Figures 2 and S1-4), particularly for larger clusters, reducing the risk of bias in spectroscopic calculations. Taken together, these findings support the use of guided, continuous DMC wave functions and probability amplitudes for GSPA analysis as a practical means to reduce computational cost. Despite the simple functional form of the q-SPC/Fw surface, it remains a suitable testing ground for evaluating the GSPA method’s ability to capture anharmonic effects.

**Figure 1:**
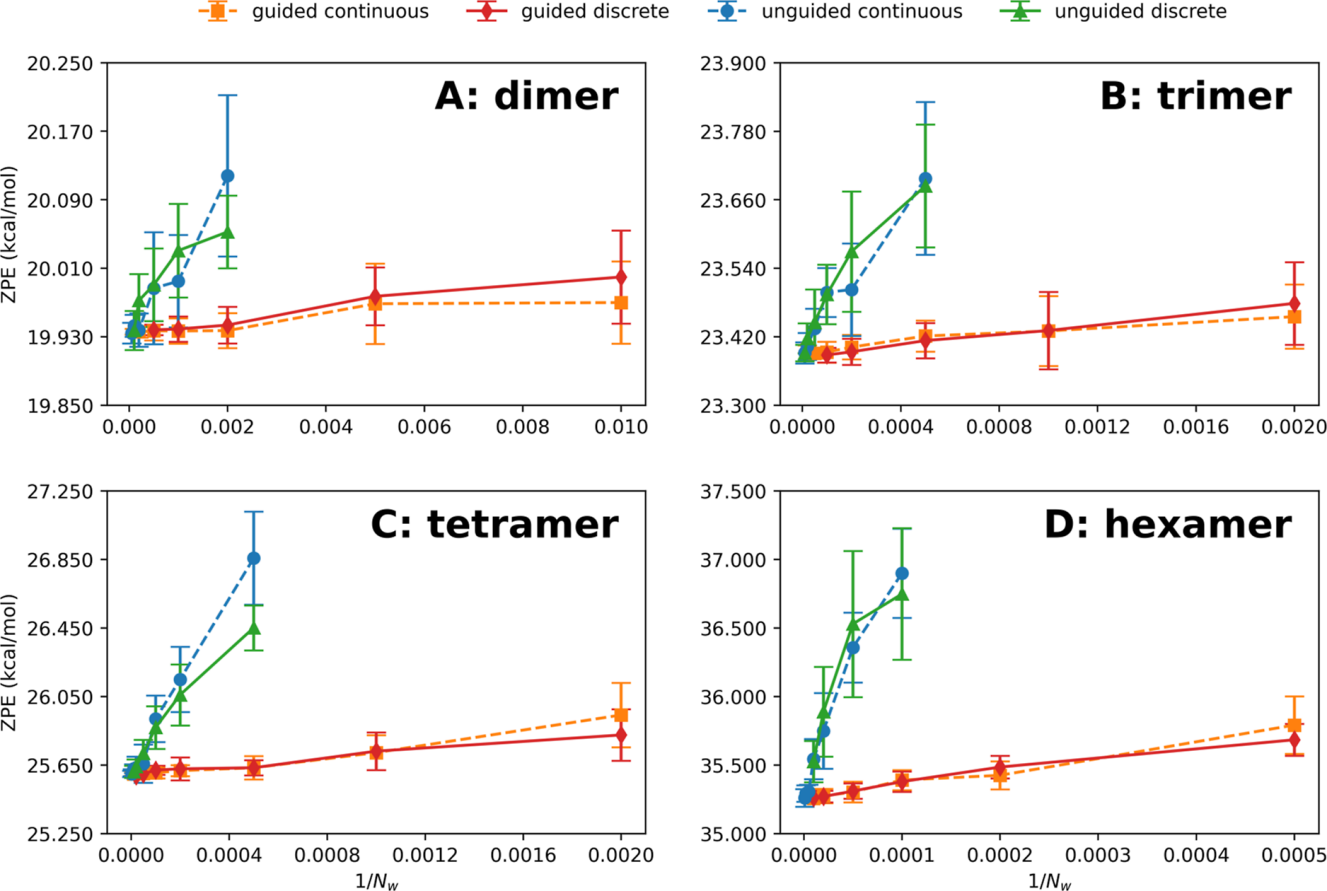
ZPE from DMC sampling versus 1/*N*_w_, compared across unguided and guided DMC with continuous and discrete weighting schemes for the (A) dimer (*N*_w_ = 100–20,000), (B) trimer (*N*_*w*_ = 500–50,000), (C) tetramer (*N*_w_ = 500–500,000 for continuous weighting, *N*_w_ = 2, 000–100,000 for discrete weighting), and (D) hexamer (*N*_w_ = 2,000–2,000,000 for continuous weighting, *N*_w_ = 2,000–100,000 for discrete weighting); *N*_DMC_ = 10. Wave functions were initialized in the most stable isomer for all clusters except for hexamer simulations with *N*_w_ ≥ 100,000, where the wave functions were delocalized equally across the first seven isomers. Numerical values are reported in Table S3.

**Figure 2:**
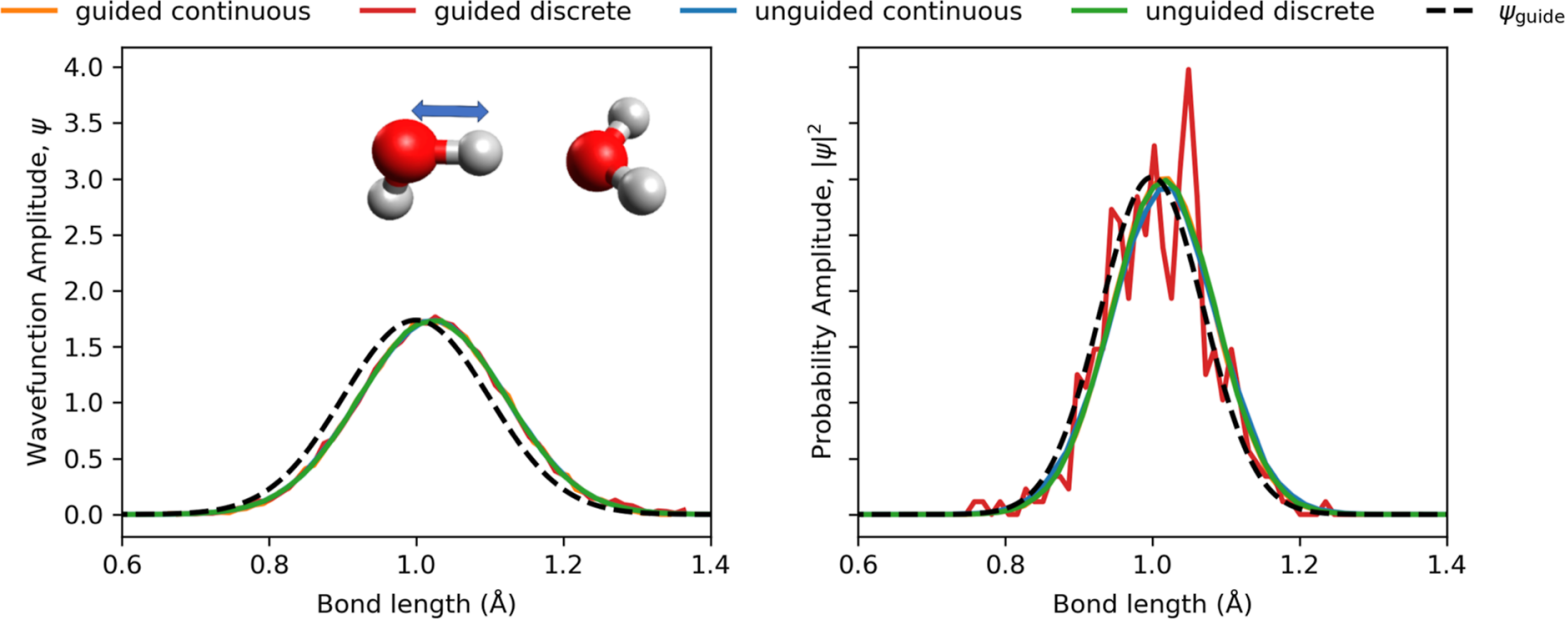
Water dimer: DMC wave function (left) and ground-state probability (right) along the H-bonded OH length of the donor molecule, compared across unguided, guided DMC with discrete and continuous weighting; *N*_w_ = 10,000, *N*_DMC_^c^ = 10, τ_DW_ = 1000, *N*_DW_ = 3.

To assess the impacts of the choice of the potential on the accuracy of predicted properties, Table S4 compares computed dissociation energies on q-SPC/Fw to previous work that computed ZPEs of many of these clusters.^14,16,19^ q-SPC/Fw model systematically overestimates dissociation energies relative to MB-Pol due to the lack of many-body effects and charge polarization, which also leads to isomers with different geometries than those on MB-pol, as summarized in Figures S5 and S6. The isomers on q-SPC/Fw PES feature much more linear O···H–O angles (i.e., more directed hydrogen bonds) and adopt more flat geometries due to the Coulombic model. This preference is observed in the hexamer as a more flat GM Book structure, which is qualitatively consistent with the Book minimum found by Shields et al. at the MP2/CBS-e level,^48^ though noticeably more opened up, and the lowest local minima (LM1) cyclic Chair Ring with *S*_6_ symmetry, also consistent with ref 48, emerge as the most stable isomers, rather than the three-dimensional Prism and Cage structures favored on MB-Pol.^14^ On q-SPC/Fw, the Cage is unstable, while the Prism fails to form as a closed structure and instead opens up, reflecting the linear hydrogen-bonding preference enforced by dominant Coulombic interactions. For each cluster studied, the wave function localizes completely or predominantly within the global minimum (GM), as the GM lies significantly lower in potential energy than all other isomers, when present.

For the hexamer, the GM remains geometrically similar to certain other isomers, leading to a small apparent leak of wave function amplitude (0.08%) when classified via energy minimization (Table 1). As long as the GM has significant initial amplitude, this leakage pattern is independent of the initial distribution of wave function amplitude across isomers, and remains statistically identical with longer simulation times (Figure S6). These observations suggest that the true wave function is fully localized in the GM, with the minor apparent leakage arising only near basin boundaries as defined by the energy minimization algorithm. Upon descendant weighting, the probability amplitude more predominantly localizes at the GM, especially at longer τ_DW_ (Figure S7). A more detailed discussion of localization of the hexamer is included in the Supporting Information.

**Table 1:** Water Hexamer Distribution of Wave Function Amplitude from Guided DMC^a^

weighting scheme	LM0/%	LM1/%	LM2/%	LM5/%
continuous	99.92(5)	0.0005(5)	0.07(5)	0.001(1)
discrete	99.92(5)	0.0004(3)	0.08(5)	0.001(1)

a *N*_DMC_ = 10, *N*_w_ = 100,000, τ = 50,000.

Furthermore, we found that classifying DMC walkers via energy minimization can overestimate the extent of isomerization events on the q-SPC/Fw PES, and thus underestimate the degree of wave function localization within the GM basin. The small fraction of walkers assigned to LM1, LM2, and LM5 (Table 1) are found to be geometrically similar to those assigned to the GM. Figure 3 compares the O–O distance distributions for ensembles of walkers initialized in the GM, fully equilibrated, and the classified as isomers according to the energy minimization procedure. The LM2-classified walkers of these ensembles exhibit a nearly identical distribution of O–O distances to that of GM (peak at 2.9 Å), rather than the 4.6 Å peak expected for true LM2 localization. This suggests that the walkers in question likely reside in the GM basin but were misassigned due to the energy minimization procedure pulling them across ambiguous basin boundaries. Repeating the analysis using a conjugate gradient method instead of BFGS yielded similar results. In high-dimensional configuration space, the boundaries between basins of attraction, defined by energy minimization as in Mallory’s method,^14^ become increasingly ill-defined, especially when walkers deviate from minimum-energy pathways. For the purpose of interpreting DMC wave function amplitudes, a classification scheme based on geometric similarity or chemically meaningful coordinates may provide a more intuitive and physically relevant description.

**Figure 3:**
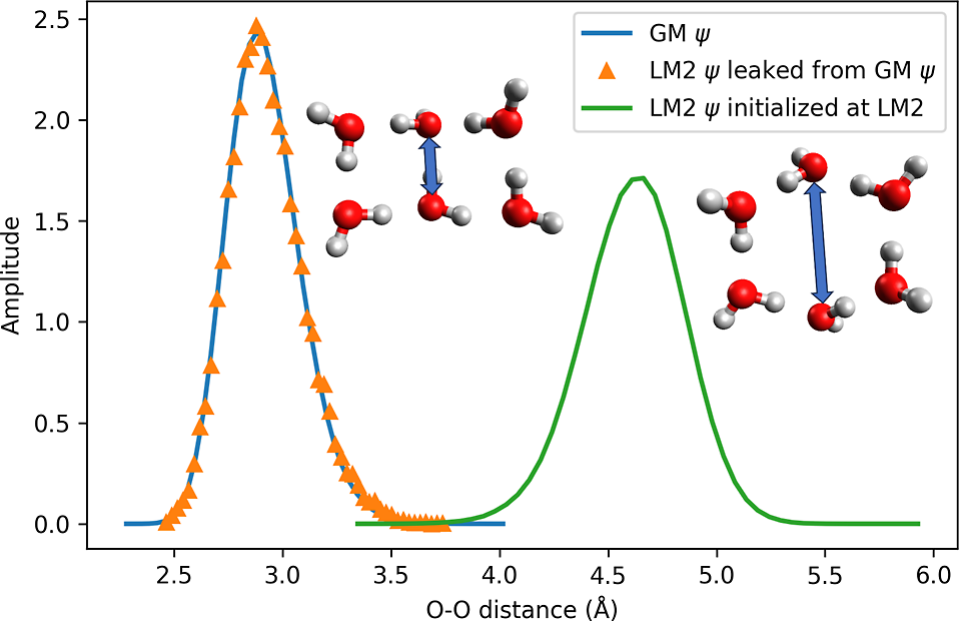
Comparison of O–O distance distributions that corresponds to the GM-to-LM2 ring-opening coordinate. Blue and orange curves correspond to walkers classified as GM and LM2, respectively, from DMC simulations with delocalized initialization (see Table 1). The green curve shows a separate guided DMC simulation initialized and localized in LM2 (*N*_w_ = 50,000). All simulations used discrete weighting.

The observed localization of the wave function in a single isomer simplifies the application of the GSPA approach. Although this localization does not preclude double- well behavior along vibrational coordinates associated with atomic permutation, previously shown to cause frequency overestimation in GSPA,^12^ we find that all forms of permutation are energetically unfavorable (Table S2). The resulting permutation probability is below 0.04% across all clusters studied. Thus, both isomeric and permutational localization elimi- nates the need for explicit walker sorting prior to spectroscopic calculations.

### Convergence of Probability Amplitudes

In addition to wave function convergence, GSPA spectroscopic analyses rely on a well-converged probability amplitude. Based on the two systematic benchmarking analyses described subsequently, we adopted τ_DW_ = 1000 for all spectroscopic predictions.

First, we monitored the convergence of the standard deviation (i.e., geometric width) of the probability amplitude along coordinates associated with various vibrational motions. The |ψ|^2^ width metric is relevant, as the shape of the probability amplitude along internal coordinates directly governs mode extraction and frequency estimation. The convergence behavior, mostly monotonic, is illustrated in Figure 4. In general, delocalized motions exhibit more slowly converging |ψ|^2^ widths, particularly flexible ring motions in the tetramer and hexamer. τ_DW_ = 1000 provides improved convergence across many types of vibrational motions and cluster sizes, as quantified in Table S5.

**Figure 4:**
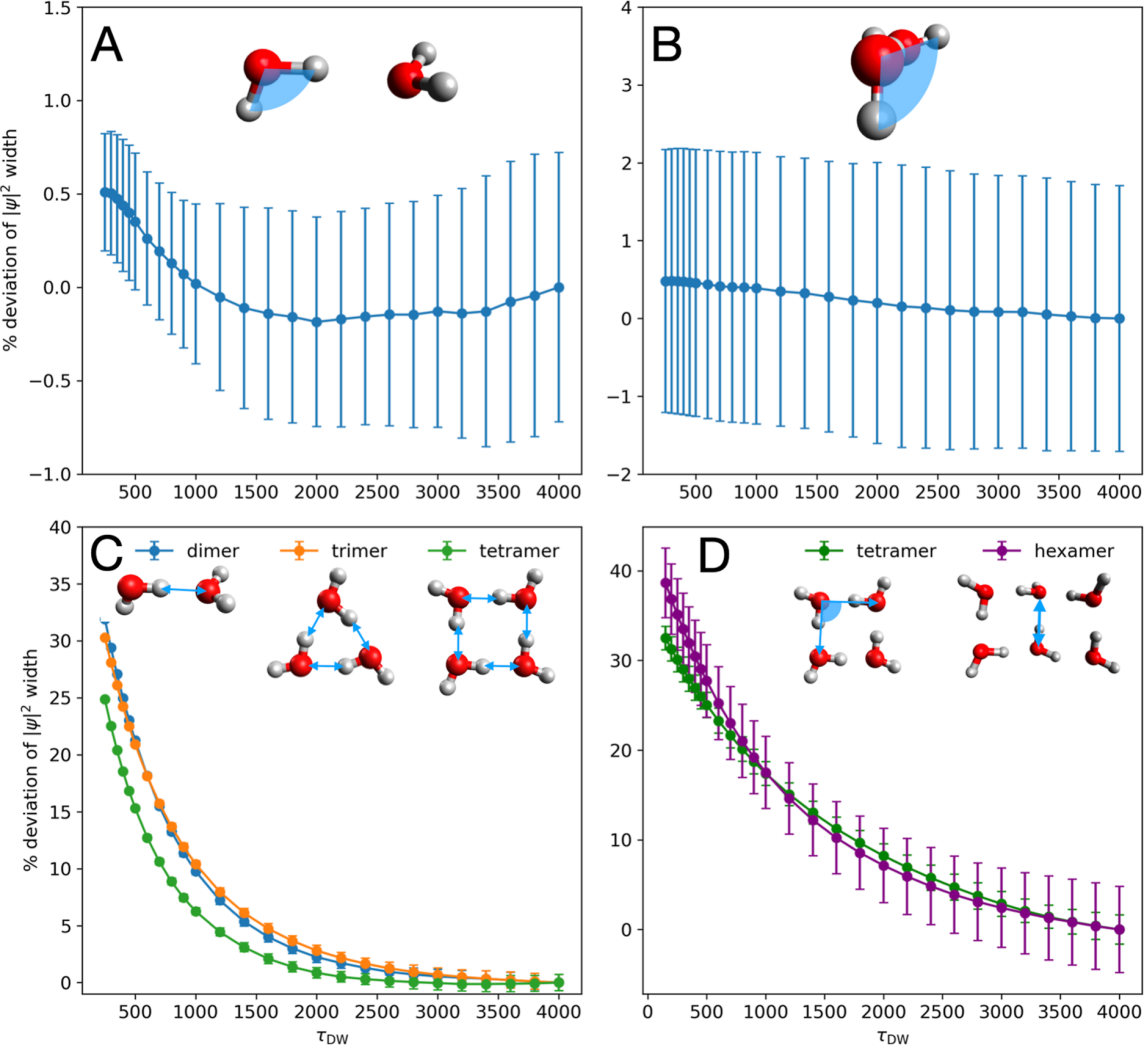
Convergence of |ψ|^2^ width with respect to τ_DW_ projected on selected internal coordinates in water clusters of different sizes. (A) dimer donor HOH angle; (B) dimer HO···OH dihedral angle; (C) dimer/trimer/tetramer H-bonding distance (defined as the intermolecular O···H distance). (D) Tetramer ring distortion (defined as the O···O···O angle) and hexamer ring-opening coordinate (defined as the O···O distances, as in Figure 3). Deviations are computed as the percent differences from widths from τ_DW_ = 4000. Means and standard deviations are computed over *N*_DMC_ = 10.

While a larger τ_DW_ yields a more converged probability distribution, it also introduces greater statistical noise, as reflected in the error bars of Figure 4. This issue was previously reported by McCoy,^10^ who attributed it to the distribution of τ_DW_ becoming increasingly localized onto fewer walkers. As shown in Figure 5, as τ_DW_ increases, the fraction of walkers with numerically nonzero *W* ^DW^ decreases nearly exponentially, with the decay rate linearly correlated with the cluster size. Thus, the effective sample size for expectation value calculations shrink with respect to both τ_DW_ and cluster size, explaining the increasing statistical noise observed in Figure 4. Despite this trend, Table S6 shows that τ_DW_ = 1000 maintains a reasonable effective sample size across all clusters, supporting its use as a practical balance between convergence and statistical reliability.

**Figure 5:**
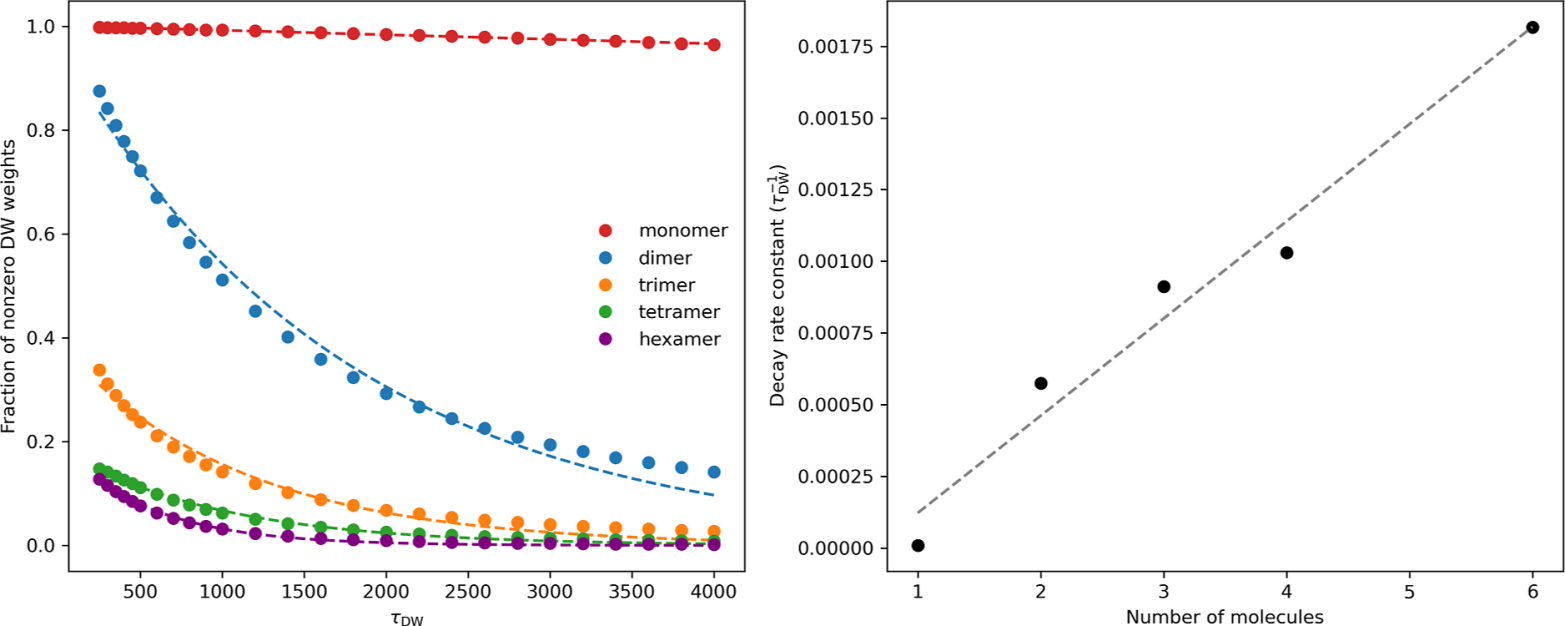
Left: fraction of walkers with (*W* ^DW^ > 10^–7^) as a function of τ_DW_ for the monomer (*N*_*w*_ = 5,000), dimer (10,000), trimer (50,000), tetramer (100,000), and hexamer (100,000). *N*_DMC_ = 10. Solid markers represent simulation results; dashed lines indicate exponential fits used to extract decay rate constants. Right: extracted exponential decay rate constants (in units of *τ*_DW_^–1^) vs the number of water molecules in each cluster. A linear fit (slope: 5.38 × 10^–4^ and *R* ^2^ = 0.993) demonstrates a strong size dependence.

### Evaluating Coordinate Selection Methods on the Water Dimer

In this section, we compare spectroscopic results predicted from the GSPA methodology across standard SVD-based and chemically informed coordinate selections, each applied to two redundant bases: ${\mathrm{r⃗}}_{N^{2}}$ containing only OH bond lengths, HOH angles, and intermolecular pairwise atomic distances, and ${\mathrm{r⃗}}_{N^{3}}$ , an augmented set that additionally includes all intermolecular angles. These comparisons are performed on a water dimer data set with *N*_DMC_^c^ = 5, *N*_w_ = 10,000, and τ_DW_ = 1000.

Comparisons with experiment are made with the IR predissociation spectra of gas phase clusters collected by Coker et al.,^49^ Page et al.,^50^ and Huang and Miller,^51^ and Zhang et al.^52^ and in inert-gas (He) clusters.^53^ With the standard SVD approach with the ${\mathrm{r⃗}}_{N^{2}}$ , the spectroscopic predictions for the OH stretches and HOH bends are in only modest agreement with measurements with many of the modes red-shifted from experimental observations and are sensitive to the initial redundant basis set. Increasing the basis to ${\mathrm{r⃗}}_{N^{3}}$ results in worse agreement. The free OH stretch has been observed consistently between 3730 ^49–51,53^ and 3732 ^52^ cm^–1^. The standard SVD approach with ${\mathrm{r⃗}}_{N^{2}}$ predicts the mode at 3569 cm^–1^. The acceptor symmetric stretch has been assigned to modes observed at 3600,^49–51^ 3603,^52^ 3654 ^53^ cm^–1^, with the standard SVD ${\mathrm{r⃗}}_{N^{2}}$ prediction at 3631 cm^–1^. The acceptor asymmetric stretch varies across experimental conditions from 3714,^50^ 3722,^49^ 3739,^51^ to 3748 ^53^ cm^–1^ and the standard SVD ${\mathrm{r⃗}}_{N^{2}}$ mode is predicted at 3675 cm^–1^. The donor hydrogen bond stretch has been observed at 3532,^49^ 3545,^50^ 3530,^51^ 3549,^52^ and 3597 ^53^ cm^–1^ and the standard SVD ${\mathrm{r⃗}}_{N^{2}}$ prediction of 3569 cm^–1^. The GSPA method leads to fully coupled vibrational motions that can be classified by the in-phase and out-of phase combinations of the symmetric stretching and donor asymmetric stretching combined with acceptor stretching. This is in contrast to previous assignment descriptions^49–52^ that assign modes using local mode descriptions, as well as the local monomer approach. The highly coupled nature of the standard SVD ${\mathrm{r⃗}}_{N^{3}}$ make it challenging to match to experiment, but overall they are considerably red-shifted from observations (Table 2).

**Table 2:** Intramolecular Vibrational Modes of the Water Dimer Using Standard Coordinate Selection^a^

${\mathrm{r⃗}}_{N^{2}}$ coordinates, CIF_intra_: –0.138				
mode assignment	${\mathrm{\nu ̃}}_{1⃗\to 0}\;\left({cm}^{-1}\right)$	Δ⟨*V*⟩_1→0_	Δ⟨*T*⟩_1→0_	χ_*l*_
A and D bends (in)	1395	695	700	0.76
A and D bends (out)	1406	710	696	0.82
H-bonded OH stretch	2434	1443	991	0.36
A sym. and D free OH stretch (in)	3569	1806	1764	0.74
A sym. and D asym. stretch	3631	1802	1829	0.87
A asym. and D asym. stretch	3675	1815	1859	0.81
${\mathrm{r⃗}}_{N^{3}}$ coordinates, CIF_intra_: –0.390				
A bend	1236	611	625	0.47
A and D bend (in)	1251	653	598	0.50
H-bonded OH and A sym. stretch (out)	2209	1309	899	0.33
A and D sym. stretch (in)	3436	1729	1706	0.63
A single OH and D free OH stretch	3541	1753	1788	0.67
A asym. and D free OH stretch	3562	1774	1788	0.60

a All energies and frequencies are reported in cm^–1^. Mode assignments follow reverse mapping. “A” and “D” denote acceptor and donor molecules, (in) and (out) signify in-phase or out-of-phase combinations.

To determine the physical origin of the dependency on and subsequent redshift of the starting redundant basis set, we assess whether the final vibrational basis *v⃗* draws proportionally from intramolecular and intermolecular descriptors in the redundant set. We analyze the (3*N* – 6) × *M* transformation matrix

32 $$T=P^{T}U^{T}$$

which maps redundant internal coordinates *r⃗* to vibrational coordinates *v⃗*. Partitioning the columns of **T** into *K* intramolecular and *M*–*K* intermolecular descriptors, we define the total intramolecular character as the cumulative contribution of intramolecular descriptors across all vibrational modes

33 $$\chi_{intra}^{total}=\sum_{i=1}^{3N-6}\;\sum_{j=1}^{K}T_{ij}^{2}$$

The value of χ_intra_^total^ reveals to what degree all of the intramolecular redundant internal coordinates are incorporated into the vibrational modes. We deem a basis chemically balanced if the value is equal to *K*. If intramolecular coordinates are underrepresented, χ_intra_^total^ will be less than *K*. The deviation from this expectation is quantified by the intramolecular character imbalance factor (CIF_intra_)­

34 $${CIF}_{intra}=\frac{\chi_{intra}^{total}-K}{K}$$

where a value of zero indicates perfect proportionality in descriptor contributions.

We further partition the intramolecular descriptors into bond and angle components, where *K*_bond_ is equal to the number of OH covalent bonds and *K*_angle_ is the number of HOH angles. Their total characters and associated imbalance factors are defined as

35 $$\chi_{bond}^{total}=\sum_{i=1}^{3N-6}\;\sum_{j\in bond}T_{ij}^{2},{CIF}_{bond}=\frac{\chi_{bond}^{total}-K_{bond}}{K_{bond}}$$

36 $$\chi_{angle}^{total}=\sum_{i=1}^{3N-6}\;\sum_{j\in angle}T_{ij}^{2},{CIF}_{angle}=\frac{\chi_{angle}^{total}-K_{angle}}{K_{angle}}$$

Importantly, all CIFs are invariant under the action of **P** ^*T*^, since **P** ^*T*^ is an orthonormal rotation. Any imbalance observed after PCA therefore reflects an imbalance already present in the reduced coordinate basis **U** ^*T*^. The CIFs thus serve as chemically meaningful diagnostics for evaluating the quality of coordinate selection. In the chemically informed construction, all CIFs are identically zero by design.

To assess the overall coupling across descriptor types, we define a mixing fraction that quantifies the extent to which intermolecular descriptors contribute to intramolecular-like modes

37 $$f_{inter\to intra}=\frac{\sum_{i=1}^{K}\;\sum_{j=K+1}^{M}T_{ij}^{2}}{\chi_{intra}^{total}}$$

Here, *i* indexes the first *K* vibrational modes and *j* spans the intermolecular descriptors. A nonzero value of *f*_inter→intra_ indicates that collective intermolecular distortions contribute to localized intramolecular motions, a physically realistic effect. For example, displacing an OH bond should perturb nearby intermolecular distances. A well-constructed vibrational basis should therefore allow moderate cross-descriptor mixing.

To assess the physical nature of each vibrational mode, we define a mode-specific character index, χ_*l*_, which measures the contribution from descriptors associated with the expected motion type of mode *l*. For example, for a stretching mode, χ_*l*_ is defined as the total contributions from OH bond descriptors

38 $$\chi_{l}=\sum_{j\in bond}T_{lj}^{2}$$

For bending modes, the sum is instead taken over HOH angle descriptors, and for intermolecular modes, over intermolecular descriptors. The value of χ_*l*_ ∈ [0, 1] reflects how faithfully the mode is localized in its intended coordinate type, with values near 1 indicating a physically well-isolated mode and lower values suggesting mixing with other types of motion.

The intramolecular mode predictions show complication with choices in the initial coordinate selection. Under the standard SVD method, the inclusion of intermolecular angles in the redundant basis set changes the character of intramolecular vibrational motions and shifts their frequencies significantly. As shown in Table 2, changing the basis from ${\mathrm{r⃗}}_{N^{2}}$ to ${\mathrm{r⃗}}_{N^{3}}$ alters both the frequencies and mode assignments for the bending and stretching motions, and sometimes the order of modes. Notably, the frequencies of the lowest OH stretch and the two bending modes are lowered by more than 150 cm^–1^. By contrast, the chemically informed SVD method results in mode predictions that are mostly invariant with respect to the redundant basis (Table 3); the in-phase A and D symmetric stretch is lowered by only 1 cm^–1^, while all other frequencies and assignments remain unchanged.

**Table 3:** Intramolecular Vibrational Modes of the Water Dimer Using Chemically-Informed Coordinate Selection Comparing ${\mathrm{r⃗}}_{N^{2}}$ and ${\mathrm{r⃗}}_{N^{3}}$ ^a^

	${\mathrm{\nu ̃}}_{1⃗\to 0}\left({cm}^{-1}\right)$ )		χ_*l*_	
mode assignment	${\mathrm{r⃗}}_{N^{2}}$	${\mathrm{r⃗}}_{N^{3}}$	${\mathrm{r⃗}}_{N^{2}}$	${\mathrm{r⃗}}_{N^{3}}$
in-phase A and D bend	1416	1416	0.84	0.75
out-of-phase A and D bend	1423	1423	0.85	0.91
in-phase A and D sym. stretch	3578	3577	0.85	0.94
out-of-phase A and D sym. stretch	3608	3608	0.74	0.79
A single OH and D asym. stretch	3646	3646	0.89	0.92
A and D asym. stretch	3676	3676	0.81	0.88

a CIF_intra_ is zero by construction.

The sensitivity of the high-frequency mode predictions from standard SVD method to the choice of redundant basis (Table 2) stems from an imbalanced coordinate selection that disproportionately favors intermolecular descriptors. The negative CIF_intra_ for standard SVD on ${\mathrm{r⃗}}_{N^{2}}$ indicates an underrepresentation of intramolecular motions. Notably, a low bond character and a loss of virial behavior for the lowest-frequency stretching mode are observed, which indicate large anharmonicity and explain its unphysically low frequency. Expanding the basis to ${\mathrm{r⃗}}_{N^{3}}$ worsens the redshifts observed in ${\mathrm{r⃗}}_{N^{2}}$ , explained by the more negative CIF_intra_, lowered individual bond and angle characters, and further loss of virial behaviors, as shown in Table 2). In contrast, the chemically informed selection maintains balanced mode representations under either ${\mathrm{r⃗}}_{N^{2}}$ or ${\mathrm{r⃗}}_{N^{3}}$ , as the CIF_intra_ remains zero by construction. As shown in Table 3, switching from ${\mathrm{r⃗}}_{N^{2}}$ to ${\mathrm{r⃗}}_{N^{3}}$ yields intramolecular frequencies that are consistent to within 1 cm^–1^. Bond and angle characters remain high. Virial behavior is preserved across all stretching modes (Table S11). Furthermore, although the chemically informed selection explicitly separates the intramolecular and intermolecular descriptor spaces during redundancy reduction, we observe a moderate *f*_inter→intra_, indicating that PCA-based vibrational coordinate extraction reintroduces mixing between descriptor types. Moderate mixing is also observed across other clusters studied (Table S13).

While specific mode identities may shift slightly at larger τ_DW_ (for example, under the chemically informed method, the highest-frequency mode changes from an “A and D asymmetric stretch” to an “A asymmetric and D free OH stretch” as τ_DW_ increases from 1000 to 4000), the overall trends discussed above remain consistent. The chemically informed SVD continues to exhibit greater self-consistency across different redundant bases, preserving balanced intramolecular vibrational mode representations more reliably than the standard SVD.

Unlike the complication of intramolecular vibrations, mode assignments were found to be consistent for low-frequency, intermolecular-like vibrations, across the two coordinate selection methods under either redundant basis, as shown in Table 4. For either the ${\mathrm{r⃗}}_{N^{2}}$ and ${\mathrm{r⃗}}_{N^{3}}$ , two coordinate selection methods yield highly similar intermolecular modes. This consistency confirms that the chemically informed approach preserves the physical structure of the low-frequency vibrational space. Nevertheless, for both coordinate selection methods, the low-frequency modes remain sensitive to the choice of redundant basis.

**Table 4:** Fundamental Frequencies (cm^–1^) of Intermolecular Vibrational Modes of the Water Dimer, Comparing Standard SVD and Chemically-Informed Method with ${\mathrm{r⃗}}_{N^{2}}$ and ${\mathrm{r⃗}}_{N^{3}}$ ^a^

	standard	chem-informed	standard	chem-informed	experiment^b^
basis	${\mathrm{r⃗}}_{N^{2}}$	${\mathrm{r⃗}}_{N^{2}}$	${\mathrm{r⃗}}_{N^{3}}$	${\mathrm{r⃗}}_{N^{3}}$	He-droplet
donor wag	90	90	57	59	86
acceptor oop libration	173	173	156	158	108–118
OH···O stretch	186	204	186	203	
ip libration	228	227	207	217	283
ip libration	354	333	340	314	283,296
oop libration	680	656	657	654	492,503

a Vibrational motions are assigned based on reverse mapping.

b Adapted from data in ref 54 (Licensed under CC BY-NC).

### Comparing Coordinate Selection Methods Across Different Clusters

To further evaluate the reliability and physical consistency of the GSPA method under different coordinate selection strategies, we examine both the CIFs and fundamental frequencies across increasing water cluster sizes. In particular, we focus on the lowest bending and stretching frequencies, as these serve as direct indicators of any underestimation in mode representation.

We found that the eigenvalues of the mass-weighted covariance matrix (eq 8) rank vibrational modes more consistently than their frequencies. Across all τ_DW_ ≥ 1000 and all cluster sizes using both standard and chemically informed SVD methods, eigenvalue-based sorting reliably preserves mode identity. This is especially important for standard SVD, where underestimated stretching modes may appear below bending modes in frequency, but remain correctly ranked by eigenvalues.

Figure 6 shows CIF_bond_, CIF_angle_, and CIF_intra_ values obtained from the standard SVD method, evaluated under ${\mathrm{r⃗}}_{N^{2}}$ and ${\mathrm{r⃗}}_{N^{3}}$ . We observe constantly more negative CIFs as the number of molecules increases. This trend is expected, as the number of intermolecular descriptors (i.e., pairwise intermolecular atomic distances) grows quadratically with system size, gradually overwhelming the linearly scaling intramolecular descriptors. Expectedly, the imbalance is further exacerbated by the inclusion of intermolecular angles in the redundant basis. These trends correlate strongly with the underestimations in the predicted fundamental vibrational frequencies, as reported in Table 5.

**Figure 6:**
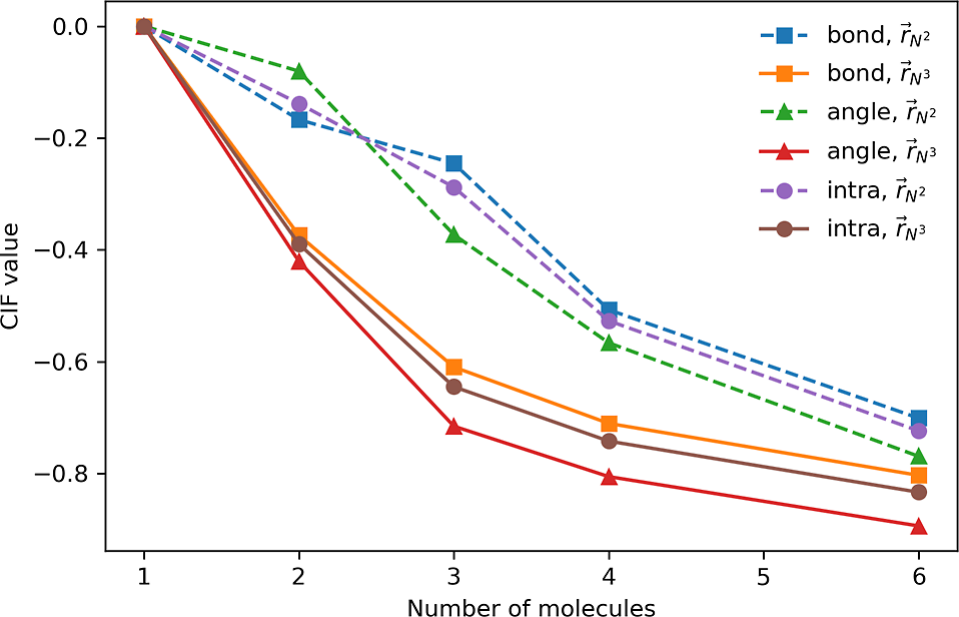
Comparison of CIF_bond_, CIF_angle_, and CIF_intra_ values under ${\mathrm{r⃗}}_{N^{2}}$ and ${\mathrm{r⃗}}_{N^{3}}$ , evaluated across different cluster sizes, using the standard SVD method at τ_DW_ = 1000. Numerical values are reported in Table S12.

**Table 5:** Lowest Predicted HOH Bending and OH Vibrational Frequencies (cm^–1^) for Each Water Cluster Size Calculated Using the Standard or Chemically-Informed SVD Coordinate Selections with ${\mathrm{r⃗}}_{N^{2}}$ and ${\mathrm{r⃗}}_{N^{3}}$ ^a^

	standard SVD				chemically informed SVD			
	HOH bend		OH stretch		HOH bend		OH stretch	
cluster	${\mathrm{r⃗}}_{N^{2}}$	${\mathrm{r⃗}}_{N^{3}}$	${\mathrm{r⃗}}_{N^{2}}$	${\mathrm{r⃗}}_{N^{3}}$	${\mathrm{r⃗}}_{N^{2}}$	${\mathrm{r⃗}}_{N^{3}}$	${\mathrm{r⃗}}_{N^{2}}$	${\mathrm{r⃗}}_{N^{3}}$
dimer	1395	1236	2434	2209	1416	1416	3578	3577
trimer	1337	1331	3290	3035	1420	1418	3576	3575
tetramer	1337	1355	2953	3000	1461	1460	3539	3538
hexamer	1251	1092	1252	1450	1414	1413	3553	3552

a All values are determined with τ_DW_ = 1000.

In contrast, the chemically informed selection maintains physical accuracy and self-consistency across all cluster sizes and the two redundant bases. As shown in Table 5, the lowest HOH bending and OH stretching frequencies for all clusters show no signs of the unphysical underestimation observed in the standard SVD method. More importantly, these frequencies differ by at most 2 cm^–1^ across the two redundant bases. This high degree of consistency further underscores the reliability of the chemically informed SVD method in preserving chemically balanced vibrational mode representations across systems with increasing complexity.

### Spectroscopic Predictions and Comparisons

Simulations of the vibrational spectra of the fundamental excitations of all clusters studied were made with the extracted vibrational modes using the initial ${\mathrm{r⃗}}_{N^{2}}$ and ${\mathrm{r⃗}}_{N^{3}}$ redundant coordinate set with the standard and chemically informed coordinate selection for the SVD reduction. Given that the PES is approximate and that our focus is on methodological development of spectral predictions rather than high-accuracy spectral prediction, we investigate only fundamental transitions. On the qSPC/Fw PES, the OH local-mode stretch harmonic is 3630 cm^–1^ and the HOH bend harmonic is 1341 cm^–1^. The predicted fundamentals across all clusters lie between 3550 and 3750 cm^–1^ (stretch) and 1400 to 1600 cm^–1^ (bend), giving Δν=ν_fund_ – ν_harm_ of −80 to +120 cm^–1^ for OH and +60 to +260 cm^–1^ for the bend. Donor OH shows the largest negative Δν, free OH lies near the harmonic, and the bend blue shift is consistent with stiffening of the HOH angle in hydrogen-bonded environments. With the identified vibrational modes, spectral predictions were made using the GSPA approach first developed by McCoy and co-workers^12,28,32^ to determine the energy and intensities of the transitions. Spectral predictions were highly sensitive to the choice of the initial redundant basis across all clusters when using the standard coordinate selection approach. The vibrational spectra predicted using the standard SVD method (Figure S12) exhibit significant shifts in both low-frequency and high-frequency regions when the redundant basis is changed. In particular, in the water hexamer, some of the vibrational modes dominated by donor OH stretching character are unphysically red-shifted below 2000 cm^–1^. In contrast, bond-stretching features predicted with the chemically informed SVD (Figure 7) are stable across cluster sizes, showing that the automated, chemically informed approach places vibrational frequencies in the appropriate spectral regions with interpretability. Mode frequencies and assignments from the chemically informed SVD with the ${\mathrm{r⃗}}_{N^{3}}$ basis for all clusters are provided in Tables S8–S11.

**Figure 7:**
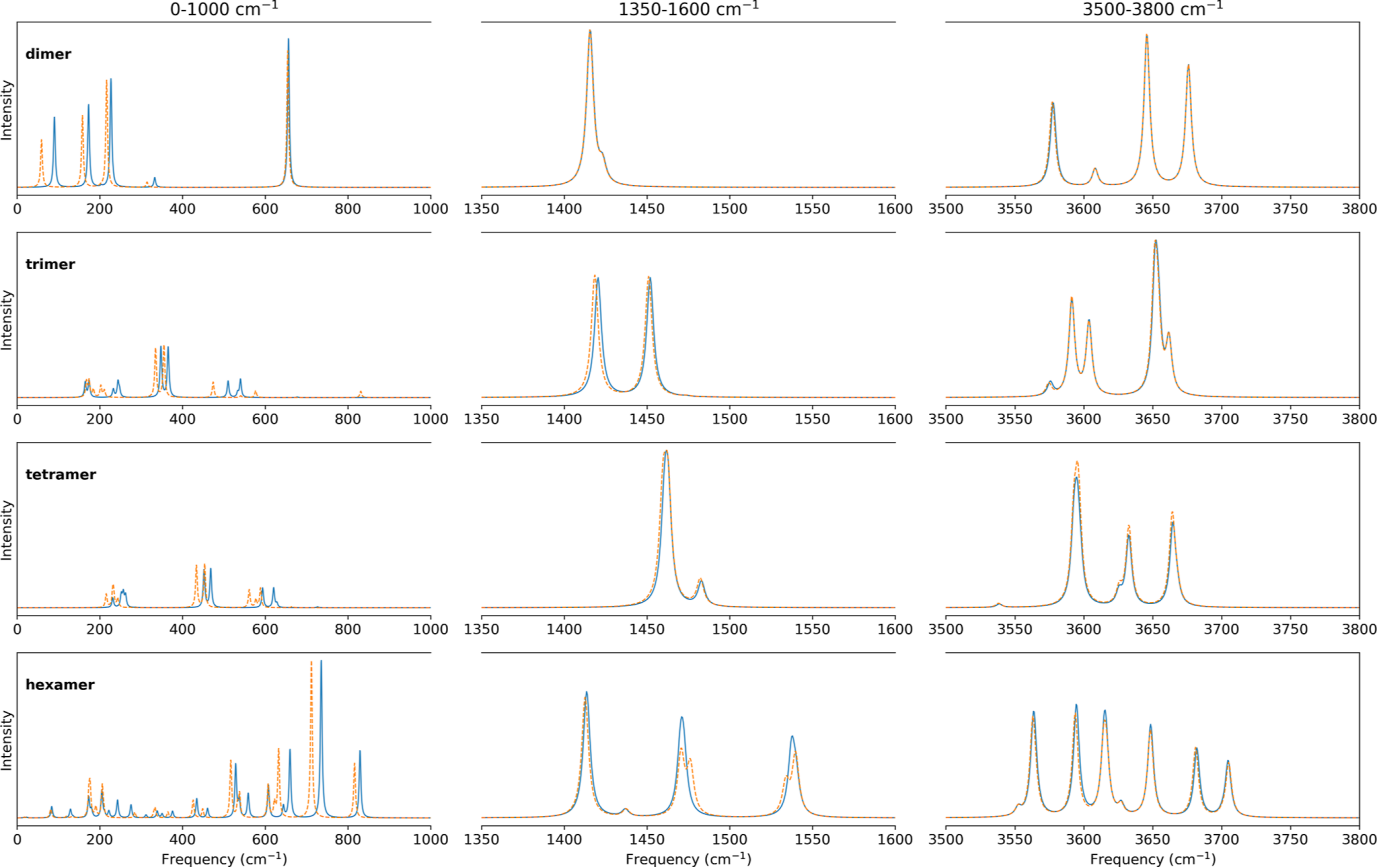
Predicted vibrational spectra obtained from chemically informed coordinate selection for the water dimer, trimer, tetramer, and hexamer, generated from ${\mathrm{r⃗}}_{N^{2}}$ (solid blue), and ${\mathrm{r⃗}}_{N^{3}}$ (dashed orange).

Across clusters, three spectral regions appear, consistent with experiment. With the chemically informed SVD, OH stretches are systematically blue-shifted but remain in a reasonable range. For the tetramer, we predict 3538 to 3668 cm^–1^ with a delocalized hydrogen bonded mode at 3596 cm^–1^; Ceponkus et al. report 3718 cm^–1^ (free OH) and 3383 cm^–1^ (hydrogen bonded).^55^ For the trimer, we predict 3651 to 3662 cm^–1^ (asymmetric) and 3575 to 3604 cm^–1^, underestimating the 3707 cm^–1^ band and overestimating the 3514 to 3516 cm^–1^ bands.^56^ In the bending region we can compare the trimer predictions to experiment. In the trimer bending region, we predict bright bands at 1451 and 1474 cm^–1^. These are red-shifted relative to the 1602 cm^–1^ HOH bend reported by Engdahl and Nelander.^56^ Schwan et al. mapped the trimer’s low-frequency spectrum with He-droplet IR.^57^ Their 86 cm^–1^ perpendicular band qualitatively matches our predicted perpendicular ring distortion at 174 cm^–1^; three bands at 150 to 215 cm^–1^ align with our 185,211 and 234 cm^–1^ modes, though missing torsional splitting in our model likely affects the latter two. We predict distinct in-plane (335 to 474 cm^–1^) and out-of-plane (541 to 831 cm^–1^) librations, whereas VPT2-based assignments used by Schwan et al. place *E* symmetry at 266 and 435 cm^–1^ and *A* near 525 cm^–1^, with no symmetric in-plane librations below 620 cm^–1^. In sum, the approach captures qualitative trends and supports assignments, but accurate vibrational frequencies will require a higher-level PES and possibly a more complete anharmonic treatment.

## Conclusions

We systematically extended and benchmarked the DMC + GSPA framework, to predict vibrational spectra for the water clusters from dimer to hexamer using the q-SPC/Fw PES. Building on prior formulations, we introduced methodological enhancements to improve the accuracy, automation, interpretability, and transferability of vibrational predictions from DMC wave functions for increasingly complex systems. Intramolecularly Guided DMC was shown to improve sampling efficiency and was essential for larger clusters like the hexamer; the use of the continuous weighting scheme is necessary for reducing numerical noise in the probability amplitude. We also examined the trade-off between the descendant weight time in the DMC + GSPA spectral prediction approach and statistical noise.

Anticipating and understanding isomerization is essential for reliable DMC sampling for larger water clusters. While basin of attraction classification is systematic, it can misclassify walkers. Classification via isomerization internal coordinates offer improved chemical interpretability.

To address the challenge of selecting a complete, nonredundant, and physically meaningful set of internal coordinates, we also developed a chemically informed SVD approach which preserves well-understood intramolecular modes. This chemically informed redundancy reduction ensures balanced coordinate selection, yields more accurate spectroscopic predictions that are invariant to the redundant basis set across all clusters studied, and maintains agreement with standard SVD in describing intermolecular vibrational features. Additionally, a reverse mapping algorithm was introduced to reconstruct Cartesian geometries from vibrational coordinates, facilitating mode assignment and interpretability. Together, these developments and benchmarking results establish a systematic workflow for vibrational spectra prediction, potentially applicable beyond water clusters. Future work will focus on extending the DMC + GSPA framework to more accurate PESs, as well as to more complex and heterogeneous molecular systems with large amplitude motions.

## Supplementary Material



## Notes

Theoretical foundation of GSPA, tabulated errors in fundamental frequency estimates from the effective harmonic approximation and GSPA for Morse oscillators of increasing anharmonicity, tabulated ZPEs computed using unguided and guided DMC, projections of probability amplitudes, permutation barrier and leakage data, a complete isomer map for the hexamer on the q-SPC/Fw potential, τ_DW_ dependence of hexamer isomer fractions, comments on wave function localization on conformational isomers, and for all water clusters studied, structural expectation values for all water clusters,and vibrational mode assignments (PDF)

## †

Department of Chemistry, University of Illinois at Urbana–Champaign, Urbana, Illinois 61801, USA
