# Supplementary material for: Enhancing Automation and Interpretability of Vibrational Spectra Predictions for Water Clusters from Diffusion Monte Carlo
Source: J Phys Chem A. 2025 Sep 16;129(38):8751–65. doi: 10.1021/acs.jpca.5c03743 (PMC12478871; doi:10.1021/acs.jpca.5c03743)
Supplement: Supplementary file 1 [file jp5c03743_si_001.pdf]

SUPPLEMENTAL INFORMATION: Enhancing Automation and Interpretability of Vibrational Spectra Predictions for Water Clusters from Diffusion Monte Carlo

Sijing Zhu[†,‡] and Lindsey R. Madison[*,†]

†*Department of Chemistry, Colby College, Waterville, Maine, 04901, United States*

‡*Present address: Department of Chemistry, University of Illinois at Urbana-Champaign, Urbana, Illinois 61801, USA*

E-mail: lindsey.madison@colby.edu

Theoretical foundation of GSPA

The ground-state probability amplitude approach (GSPA) to determining the vibrational excitation energies from a ground-state wave function was developed by McCoy and co-workers in Ref. 28, 32, and 12 of the main text. We present here an explanation of the theoretical foundation of the approach. GSPA computes the fundamental frequencies by partitioning the energy change into potential energy gap and kinetic energy gap, as shown in Equation 11, and computing both components with multiplicative operators. By construction of DMC, the ground-state wave function projected along a multiplicative observable x is defined as:

$$\psi_0(x) = \frac{\sum_j w_j \delta(x - x(\mathbf{R_j}))}{\sum_j w_j}, \tag{S1}$$

with w_j being a continuous weight of walker j. The projected probability amplitude is given by:

$$P_0(x) = \frac{\sum_j W_j^{\mathrm{DW}}\ w_j\ \delta(x - x(\mathbf{R_j}))}{\sum_j W_j^{\mathrm{DW}}\ w_j}, \tag{S2}$$

where W_j^{DW} is the descendant weight of walker j. Each $O(\mathbf{R_j})$ weighted by its local amplitude is then summed to give the expectation value of x, as shown in Equation 6.

In GSPA, the first excited state is approximated as $\psi_1(x) = f(x)\psi_0(x)$, where f_1 is the excitation polynomial defined in Equation 12. Then,

$$P_1(x) = f_1^2(x)\psi_0^2(x) \approx \frac{\sum_j W_j^{\mathrm{DW}}\ w_j\ \delta(x - x(\mathbf{R_j}))\ f_1^2(x(\mathbf{R_j}))}{\sum_j W_j^{\mathrm{DW}}\ w_j f_1^2(x(\mathbf{R_j}))}, \tag{S3}$$

which then leads to the expectation value of V in the first excited state along a specified vibrational coordinate as defined in Equation 13. The potential energy gap is obtained after subtracting by $\langle \hat{V} \rangle_0$.

Equation 14 of the main text which defines the kinetic energy contribution to the energy gap follows from the harmonic oscillator approximation in that the value can be related to multiplicative position operators x. For the harmonic oscillator the kinetic energy difference

between the ground and first excited state is:

$$\langle T \rangle_1 - \langle T \rangle_0 = \frac{\hbar^2 \alpha}{2m} \tag{S4}$$

with α defined as:

$$\alpha = \left(\frac{km}{\hbar^2} \right)^{1/2} \tag{S5}$$

For a 1D quantum harmonic oscillator in the ground state with $\langle x \rangle = 0$,

$$\langle x^2 \rangle = \frac{\hbar}{2m\omega} = \frac{1}{2\alpha} \tag{S6}$$

$$\langle x^4 \rangle = 3 \left(\frac{\hbar}{2m\omega} \right)^2 = \frac{3}{4\alpha^2} \tag{S7}$$

Thus, the difference in kinetic energy of ground harmonic oscillator state can be expressed as expectation values of second moment and fourth moment of the position operator, giving equation 14:

$$\langle T \rangle_1 - \langle T \rangle_0 = \frac{\hbar^2}{2m} \cdot \frac{\langle x^2 \rangle}{\langle x^4 \rangle - \langle x^2 \rangle^2} \tag{S8}$$

Because the true DMC states are not fully harmonic, the kinetic energy gap is approximated by Equation S8, giving equation 14 of the main text.

Table S1: **Comparison of fractional errors in fundamental frequency estimates from the effective harmonic approximation and GSPA based on DMC samplings, across increasing anharmonicity on a one-dimensional Morse oscillator potential. DMC & DW parameters:** $N^{\mathbf{c}}_{\mathbf{DMC}} = 10$, $\tau = 40{,}000$, $\tau_{\mathbf{eq}} = 20{,}000$, $N_w = 100{,}000$, $\tau_{\mathbf{DW}} = 5{,}000$. **The order of magnitude and the trend of GSPA error match with the previous study.**[1] **The anharmonicity constant is defined as a dimensionless factor,** $\hbar\omega/(4D_e)$, **where** 0.00592 **is the anharmonicity of carbon monoxide.**[?] **In the effective harmonic treatment, frequencies are estimated using** $\Delta E[1,0] = \hbar\omega = \hbar/(2\mu\sigma^2)$, **where** $\sigma^2 = \langle x^2 \rangle - \langle x \rangle^2$ **is the variance of the ground-state probability distribution.**

Anharmonic Const.	Fractional error (Eff. Harmonic)	Fractional error (GSPA)
0.0059	-0.0029	-0.00050
0.0118	0.0014	0.00080
0.0178	0.0063	-0.00080
0.0237	0.0070	0.00030
0.0296	0.0099	0.00060
0.0355	0.012	0.00050
0.0414	0.019	0.0016
0.0474	0.022	0.0038
0.0592	0.029	0.0051
0.0710	0.041	0.0064
0.0829	0.044	0.0051
0.0947	0.054	0.0076
0.1066	0.062	0.011
0.1184	0.077	0.012

Table S2: Minimum energy barriers for permutations and total permutation fractions (leakage) of the wave function amplitude and probability, evaluated across different water cluster sizes. Permutations were identified via energy minimization and Eckart alignment following guided DMC with continuous weighting, with all walkers initialized using a consistent atom labeling. Simulation parameters: $N_{\mathbf{DMC}} = 5$, $\tau = 50{,}000$, $N_w = 10{,}000$ (dimer), $50{,}000$ (trimer), and $100{,}000$ (tetramer, hexamer); $\tau_{\mathbf{DW}} = 1{,}000$, $N_{\mathbf{DW}} = 3$. These DMC datasets and parameters match those used in the spectroscopic predictions. The negligible permutation fractions—arising from high rotational energy barriers—confirm that explicit walker re-permutation is unnecessary for vibrational analyses. Energy barriers are reported in kcal/mol. MW-RMSDs, computed as the mass-weighted distances between the minimum and the transition state of permutation, are reported in Å. See Figure S11 and S8 for convention of indexing molecules for the dimer and hexamer.

Permutation	Barrier	MW-RMSD	%leakage(ψ)	%leakage($\|\psi\|^2$)
**Dimer GM**			**0.20(1)**	**0.030(6)**
A rotation (Indistinguishable)	0.0678	0.31	–	–
D rotation	2.91	0.24	–	–
A–D exchange	1.65	0.80	–	–
**Trimer GM**			**0.028(2)**	**0.0011(5)**
any monomer rotation	3.35	0.27	–	–
**Tetramer GM**			**0.015(2)**	**0.0005(4)**
any monomer rotation	4.09	0.25	–	–
**Hexamer GM**			**0.08(1)**	**0.005(3)**
molecule 4 rotation	3.40	0.55	–	–
molecule 1 rotation	NA	NA	–	–
molecule 3 rotation	3.81	0.20	–	–
molecule 2 rotation	4.35	0.19	–	–
molecule 6 rotation	4.72	0.21	–	–
molecule 5 rotation	4.21	0.24	–	–

Table S3: Comparison of ZPEs computed using unguided and guided DMC for different water clusters. Z scores significantly lower than one indicate statistical agreement.

Cluster	Unguided N_w	Guided N_w	Unguided ZPE	Guided ZPE	Z score
Dimer	20,000	10,000	19.93(1)	19.936(7)	0.13
Trimer	100,000	50,000	23.39(2)	23.398(7)	0.32
Tetramer	500,000	100,000	25.60(2)	25.60(1)	0.01
Hexamer	2,000,000	100,000	35.26(6)	35.26(3)	0.06

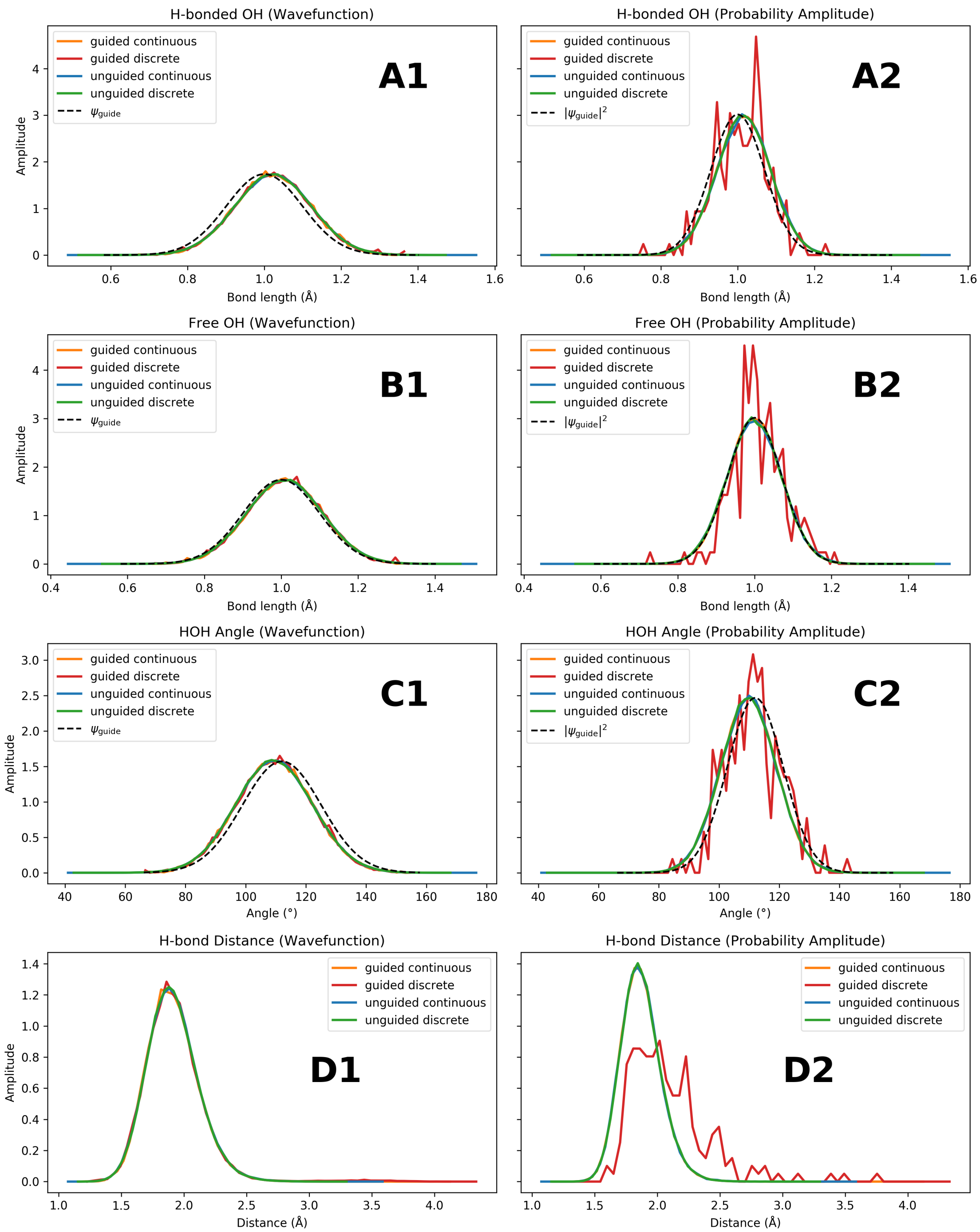

Figure S1: Water dimer: DMC wave function amplitude (1) and ground-state probability density (2) projected along the donor H-bonded OH length (A), donor free OH length (B), HOH angle (C), and H-bonding O···H distance (D). Results are shown for unguided and guided DMC with both discrete and continuous weighting. Parameters: N_w = 10,000, N^{c}_{DMC} = 10, τ_{DW} = 500, N_{DW} = 3. All four methods produce consistent wave function profiles, while discrete-weighting guided DMC exhibits noticeable statistical noise in the probability amplitude with possibly skewed distribution along intermolecular coordinates.

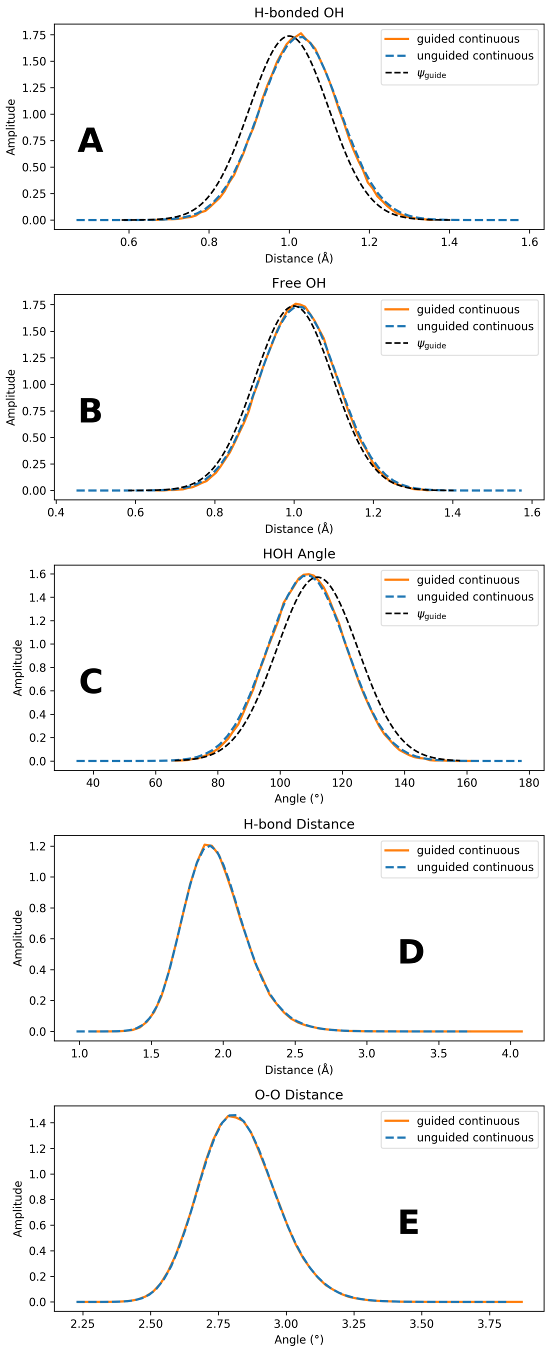

Figure S2: Water trimer DMC wave function amplitude projected along the donor H-bonded OH length (A), donor free OH length (B), HOH angle (C), H-bonding O$\cdots$H distance (D), and O-O distance (E). Results are shown for unguided (N_w = 100,000) and guided DMC (N_w = 50,000) with continuous weighting, $N^{c}_{DMC} = 5$. Two DMC methods generate agreeing wave functions.

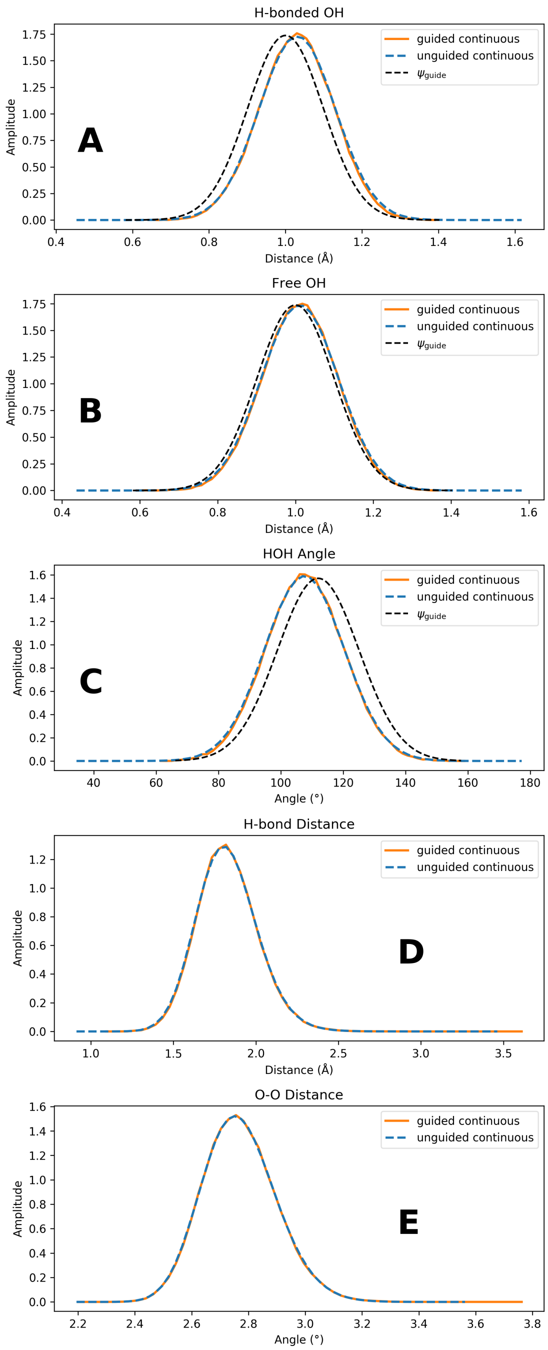

Figure S3: Water tetramer: DMC wave function amplitude projected along the donor H-bonded OH length (first row), donor free OH length (second row), HOH angle (third row), and H-bonding O$\cdots$H distance (fourth row), and O-O distance (fifth row). Results are shown for unguided (N_w = 500,000, N^{c}_{DMC} = 5) and guided DMC (N_w = 100,000, N^{c}_{DMC} = 10) with continuous weighting. Two DMC methods generate agreeing wave functions.

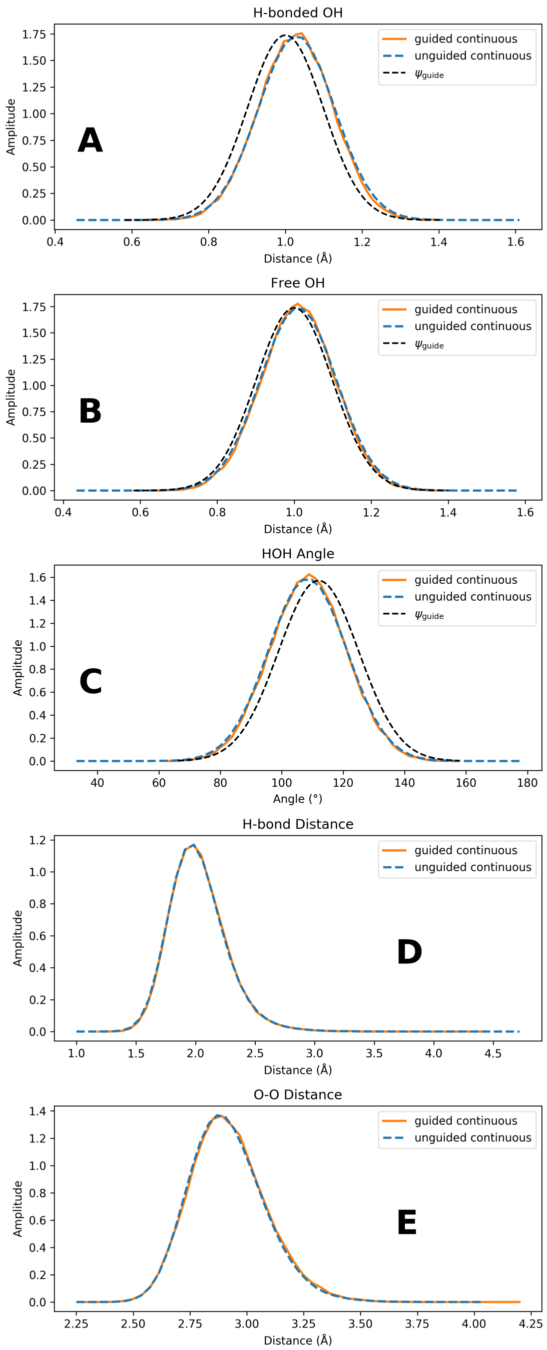

Figure S4: Water hexamer: DMC wave function amplitude projected along the donor H-bonded OH length (A), donor free OH length (B), and HOH angle (C) of molecule 2 (see Figure S8), H-bonding O$\cdots$H distance between O4 and H1B′ (D), and distance between O1 and O4 (E). Results are shown for unguided (N_w = 200,000, N^{c}_{DMC} = 3) and guided DMC (N_w = 100,000, N^{c}_{DMC} = 10) with continuous weighting. Two DMC methods generate agreeing wave functions. The slight statistical noises seen in wave function projections are not present in GSPA analysis upon DW.

Table S4: Converged dissociation energies, D_0. All values are in kcal/mol.

Cluster	$D_0((H_2O)_n) = nE_0(H_2O) - E_0((H_2O)_n)$			
	q-SPC/Fw (this work)	q-TIP4P[a]	WHBB[b]	MB-Pol[c]
Dimer	4.697(8)	4.53	3.15	3.15
Trimer	13.551(9)	—	10.694	10.85
Tetramer	23.67(1)	—	—	19.56
Hexamer	38.64(3) (book)	37.89 (cage)<br>37.24 (prism)	—	33.00 (cage)

Adapted from [a]Ref. 2 Copyright 2015 ACS [b]Ref. 3 Copyright 2011 AIP [c]Ref. 4 Copyright 2016 AIP.

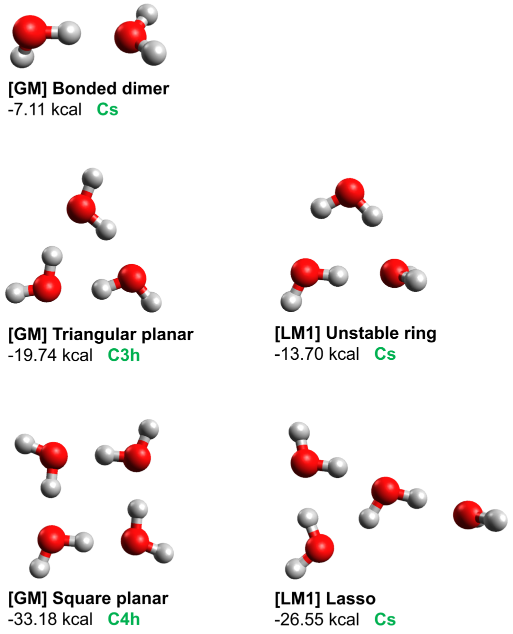

Figure S5: Geometry, potential energy, and symmetry group of the dominant isomer(s) for water dimer, trimer and tetramer.

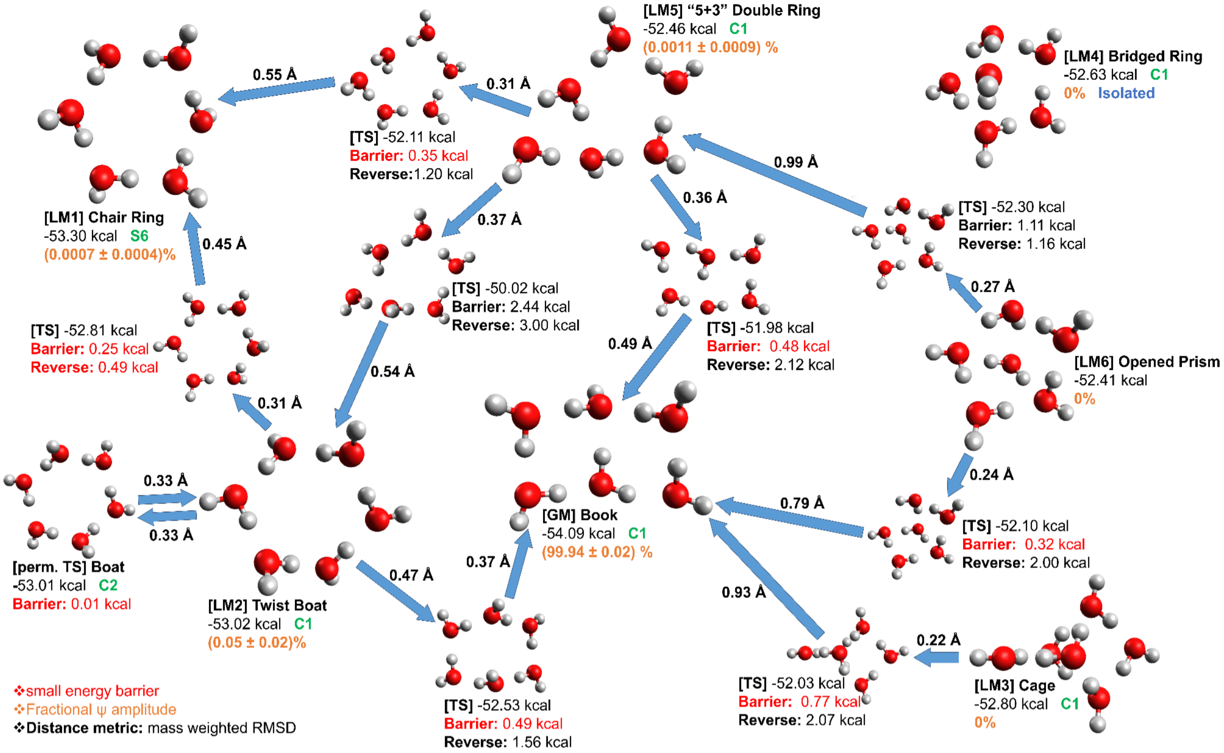

Figure S6: Energy landscape of the water hexamer explored using Metropolis Monte Carlo sampling, BFGS minimization, and CI-NEB. Up to the first seven isomers and connecting transition states (TSs) are shown. Mass-weighted RMSD was used as the distance metric to account for the distinct diffusion rates of atoms. Fractional populations were extracted from a discrete-weighting guided DMC simulation with $N_{\text{DMC}} = 10$, $N_w = 100{,}000$, $\tau = 100{,}000$, and $\tau_{\text{eq}} = 800{,}000$, where walkers were initialized equally across the seven isomers. wave function amplitude reflects both isomer stability and pathway connectivity. Although LM1 is more stable with a wider mass-weighted RMSD basin, LM2 carries greater amplitude because it is directly connected to the GM, while LM1 is reached via LM2; initializing the wave function between LM1 and LM2 reverses this trend, with analogous behavior across other hexamer isomers.

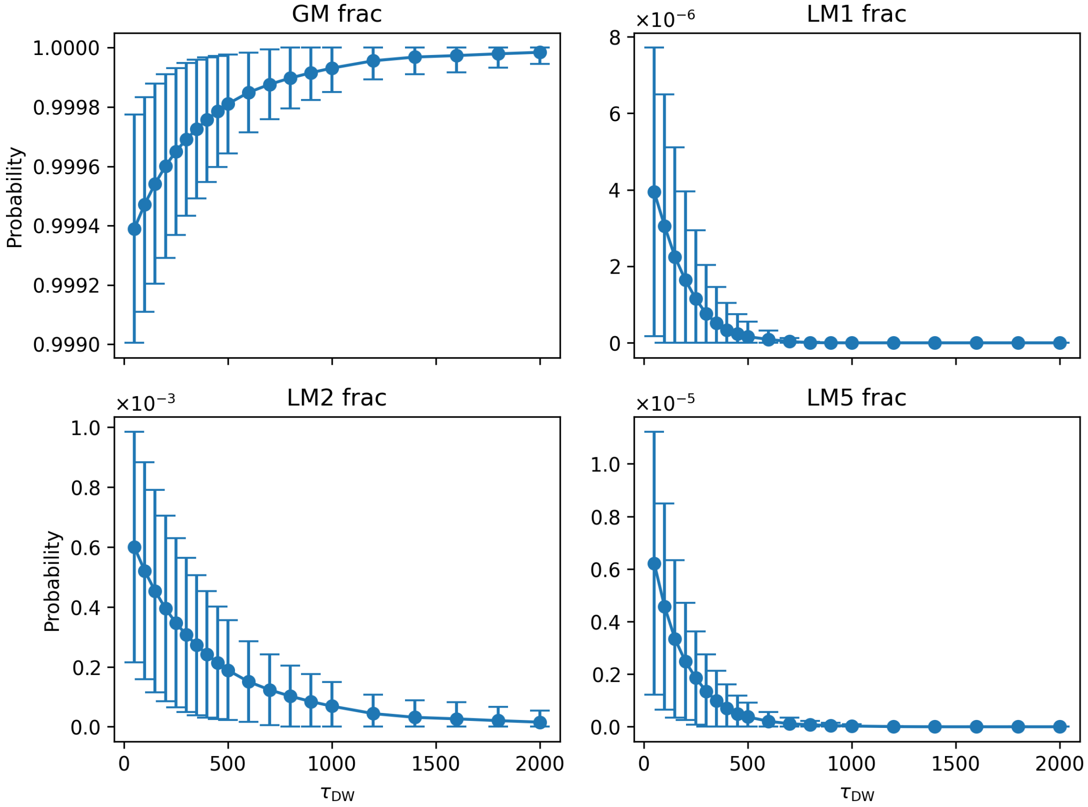

Figure S7: Hexamer isomer fractions as a function of τ_{DW} (50–2000), obtained from guided, continuous-weighting DMC simulations. Parameters: $N_{\mathrm{DW}} = 3$, $N_{\mathrm{DMC}} = 10$, $N_w = 100{,}000$, $\tau = 50{,}000$. The global minimum (GM) clearly dominates the ground-state probability, with all other isomer fractions decaying exponentially to near zero.

Wave Function Localization on Conformational Isomers

Although the DMC wave function amplitude for the dimer, trimer, and tetramer on q-SPC/Fw is entirely concentrated in the GM, the hexamer reveals a more complicated PES with multiple low-lying isomers that are closer in energy and geometry (Figure). To investigate how DMC samples this broader configurational space, we employed a large walker ensemble initialized equally across the first seven identified LM isomers and analyzed the equilibrated wave function fractions using the energy minimization-based classification scheme developed by Mallory et al.[4] We found that short-time DMC simulations ($\tau = 50{,}000$; Table 1 of the main text) yield wave function fractions that closely match those obtained from

longer simulations (τ = 100,000; population % in Figure), indicating that even moderate propagation lengths can produce consistent isomer sampling. Table 1 shows consistent results between continuous and discrete weighting schemes. Notably, wave function distribution is shaped not only by the relative stability of isomers but also by the connectivity and accessibility of pathways between them. For example, although LM1 is more stable and possesses a wider basin in terms of mass-weighted RMSD than LM2, LM2 consistently captures greater wave function amplitude due to its direct connection to the GM, which serves as a reservoir of walkers. Walkers can diffuse to LM2 directly from the GM, whereas reaching LM1 requires passing through LM2. This pattern reverses when the wave function is initialized and localized between LM1 and LM2, further confirming the role of pathway connectivity. A similar interpretation applies to the PES landscape among other isomers of the hexamer.

While the spread of vibrational amplitude across isomers may be viewed as a stationary-state behavior, DMC enables a complementary interpretation from a dynamical perspective: walker diffusion and branching equilibrate DMC wave function over τ, with most walkers localized in the global minimum and a small fraction probabilistically diffusing to connected minima. Amplitudes in regions that are separated by long, mass-weighted distances or energetically unfavorable transitions are suppressed because walkers attempting to reach these regions are typically eliminated before arrival due both to the low probability of escape from the initial basin and the elevated potential energy encountered along the transition pathway. The difficulty of fully capturing the natural isomerization results in what is termed "localization",[4] where the wave function amplitude tends to be dominated by a single isomer. The effect is similarly observed on the MB-pol PES, as reported by Mallory[4] and Lee.[5]

Given sufficient walker population, such localization may reflect inherent limitations in the DMC algorithm or simply poor initial conditions. In the latter case, when walkers are initialized in deep, high-energy isomers, a majority may remain trapped, failing to reproduce the wave function fractions obtained from delocalized initializations. Although some walkers

may leak out, they are quickly overwhelmed by those remaining in favorable regions, and the branching process reinforces this imbalance, leading to persistently unconverged yet pseudo-equilibrated sampling over long timescales. For example, in the tetramer, where the equilibrated reference energy of the Lasso isomer (Figure S5) lies 7.82 kcal/mol above the GM, simulations initialized in Lasso can sometimes remain localized for up to τ = 1,000,000 with only small fractions of the populations transitioning to other isomers. However, several trials exhibit complete isomerization back to the GM, while the reverse transition is never observed, suggesting that localization arises from initialization rather than a fundamental limitation of DMC. A similar pattern emerges in the hexamer. Walkers initialized in LM3 and LM6 localize efficiently to the GM, likely due to narrower basins or lower barriers along the escape path. In contrast, walkers initialized in LM1 and LM2 remain trapped over long propagation times. Although these results suggest that poor initialization contributes significantly to observed localization, its role cannot be fully disentangled in the absence of prohibitively long simulations.

To assess whether DMC inherently drives artificial localization or instead accurately reproduces a ground-state wave function that is actually localized due to energetic preference, we conducted a control study on the trimer GM. We constructed two equivalent permutations via a monomer rotation, separated by a barrier of 3.35 kcal/mol. wave function was initialized equally across both permutations, and interconversion remained negligible (less than 0.03%, as inferred by Table S2). Across 10 DMC simulations (N_w = 50,000 and τ = 50,000), DMC maintained comparable populations in both permutations, with average fractions of 52% $\pm$ 14% and 48% $\pm$ 14%, respectively. This result shows that in the absence of an energy difference, and with a sufficiently large walker ensemble, DMC can preserve multiple non-communicating isomers without collapsing into one. The relatively large standard deviation highlights DMC's inherent stochastic nature and sensitivity to initial imbalance, which is amplified by branching due to low interconversion probabilities. Nonetheless, the ability to preserve both isomers across trials supports that DMC does not inherently favor localization.

Table S5: Maximum percentage change (%Δ) in $|\psi|^2$ width along selected geometric coordinates within different τ_{DW} ranges.

Coordinates	%Δ (250–4000)	%Δ (1000–4000)
Monomer OH (Å)	0.24	0.15
Dimer HOH angle (°)	0.69	0.20
Dimer H-bonding distance (Å)	32	9.8
Trimer H-bonding distance (Å)	30	10
Tetramer H-bonding distance (Å)	25	6.4
Dimer HOOH dihedral (°)	0.48	0.39
Tetramer ring distortion (°)	30	17
Hexamer ring opening (Å)	35	18

Table S6: Fraction of walkers with $W^{\mathbf{DW}} > 10^{-7}$ at $\tau_{\mathbf{DW}} = 1000$, and the corresponding effective N_w that contributes non-trivially to expectation calculations.

Cluster	Fraction of Nonzero W^{DW}	N_w	Effective N_w
Monomer	0.99	5,000	4,964
Dimer	0.51	10,000	5,115
Trimer	0.14	50,000	7,105
Tetramer	0.062	100,000	6,230
Hexamer	0.032	100,000	3,150

Table S7: Expectation values of all distinct bond lengths ($\tau_{\mathbf{DW}} = 500$), bond angles ($\tau_{\mathbf{DW}} = 1000$), intermolecular O$\cdots$H (hydrogen-bonding) distances ($\tau_{\mathbf{DW}} = 4000$), and intermolecular O$\cdots$O distances ($\tau_{\mathbf{DW}} = 4000$) for the water dimer, trimer, and tetramer; $N_{\mathbf{DW}} = 3$, $N_{\mathbf{DMC}} = 10$, $N_w = 5{,}000$ (monomer), 10,000 (dimer), 50,000 (trimer), and 100,000 (tetramer).

**Cluster / quantity**	**Expectation value**
Monomer	
OH length (Å)	1.0009(4)
HOH angle (°)	111.99(4)
Dimer	
Donor Free OH	0.9992(1)
Donor H-bonded OH	1.0152(3)
Acceptor Free OH	1.0036(2)
Donor HOH angle (°)	109.86(6)
Acceptor HOH angle (°)	110.57(5)
Intermolecular O$\cdots$H (Å)	1.833(1)
Intermolecular O-O (Å)	2.7993(8)
Trimer	
Free OH	1.0020(2)
H-bonded OH	1.0185(3)
HOH angle (°)	109.15(4)
Intermolecular O$\cdots$H (Å)	2.722(3)
Intermolecular O-O (Å)	2.7981(7)
Tetramer	
Free OH	1.0020(1)
H-bonded OH	1.0233(2)
HOH angle (°)	107.96(7)
Intermolecular O$\cdots$H (Å)	2.991(3)
Intermolecular O-O (Å)	2.745(1)

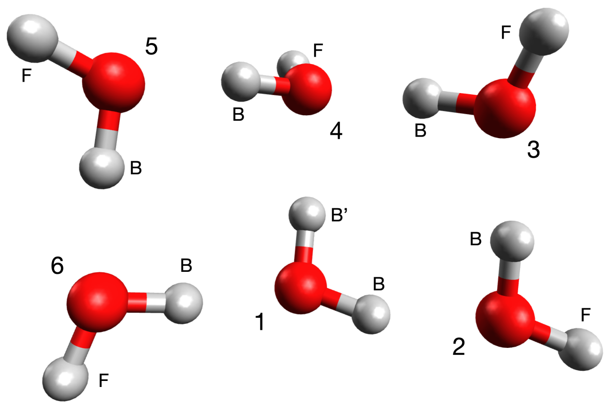

Figure S8: Minimum energy structure of the water hexamer with labeling scheme used in SI tables. "B" represents a hydrogen-bonding OH bond stretch, "F" for the free OH stretch.

Table S8: Hexamer stretching mode assignments made by visually assessing the predominant character of the optimization-based reversed mapping animations. Molecule and atomic labeling scheme follows Figure S8. $\tilde{\nu}_{1\to0}$ is the fundamental excitation frequency; $\Delta\langle V\rangle_{1\to0}$ and $\Delta\langle \mathbf{T}\rangle_{1\to0}$ are the potential and kinetic contributions, respectively. All frequencies are reported in cm^{-1}

.

Mode	$\tilde{\nu}_{1\to0}$	$\Delta\langle V\rangle_{1\to0}$	$\Delta\langle T\rangle_{1\to0}$	χ_l	Water ID number					
					1	2	3	4	5	6
36	3552	1818	1734	0.65	B	B	Sym	F	B	Sym
37	3563	1816	1747	0.63	Sym	Sym	Sym		F	Sym
38	3565	1729	1836	0.76	B	B	Asym	Sym	Asym	F
39	3594	1810	1784	0.65	B	Sym	F	F	Sym	Sym
40	3614	1777	1837	0.74	B'		Sym	B	F	F
41	3617	1863	1753	0.64	B'	F	B	B	Sym	Asym
42	3627	1755	1872	0.79	Asym	B	F	B	Asym	Asym
43	3648	1795	1853	0.88	Asym	B	B	B	B	B
44	3649	1845	1804	0.68	Sym	Asym	Asym	Sym	Asym	F
45	3681	1911	1770	0.66	B	Asym	Asym	Asym	Asym	B
46	3701	1884	1817	0.82	Sym	Asym	F		Asym	F
47	3705	1923	1782	0.71	Asym	Sym	Asym	Asym	B	B

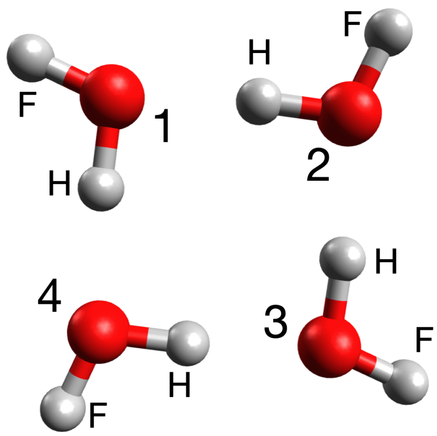

Figure S9: Minimum energy structure of the water tetramer with labeling scheme used in SI tables. “B” represents a hydrogen-bonding OH bond stretch, “F” for the free OH stretch. This isomer is a cyclic planar with C_{4h} symmetry.

Table S9: Tetramer mode assignments made by visually assessing the predominant character of the optimization-based reversed mapping animations. Labeling scheme follows Figure S9. $\tilde{\nu}_{1\to0}$ is the fundamental excitation frequency; $\Delta\langle V\rangle_{1\to0}$ and $\Delta\langle \mathbf{T}\rangle_{1\to0}$ are the potential and kinetic contributions, respectively. All frequencies are reported in cm^{-1}. The equivalency of the waters leads to in-phase and out-of-phase combinations of stretches. Bending motions are constructed as combinations of individual monomer bending motions, with relative phases indicated by the signs. Intermolecular motions are in-plane (ip) or out-of-plane (oop) libration motions, or wagging of the free OH.

Mode	$\tilde{\nu}_{1\to0}$	$\Delta\langle V\rangle_{1\to0}$	$\Delta\langle T\rangle_{1\to0}$	χ_l	Water ID number				in-phase / out-of-phase
					1	2	3	4	
					**OH stretch**				
29	3668	1809	1858	0.87	Asym		Asym	Asym	out
28	3664	1813	1851	0.76	Asym		Asym		out
27	3633	1810	1822	0.69	Asym	Asym	Asym	Asym	out
26	3626	1803	1822	0.80	Asym	Asym		Asym	out
25	3597	1798	1799	0.64	Sym	Sym	Sym		out
24	3596	1800	1795	0.61	B	B	B	B	out
23	3593	1804	1789	0.66	Sym	Sym	Sym	Sym	out
22	3538	1775	1763	0.56	Sym	Sym	Sym	Sym	in
					**Bends**				
21	1482	749	733	0.58	+	+	+	+	in
20	1463	741	721	0.65	+	−	+	−	out
19	1460	726	735	0.61	+	0	−	−	out
18	1460	720	740	0.59	+	+	−	−	out
					**Intermolecular**				
17	665	381	284	0.65	oop	oop	oop	oop	in
16	589	398	191	0.70	oop	oop	oop	oop	in
15	577	378	199	0.64	oop		oop		in
14	561	374	187	0.67				oop	
12	454	237	216	0.94	ip	ip	ip	ip	out
11	434	217	217	0.93	ip	ip	ip	ip	out
7	243	141	102	0.76	wag		wag		in
6	233	132	102	0.78			wag	wag	in
5	231	153	78	0.72		ring distortion			

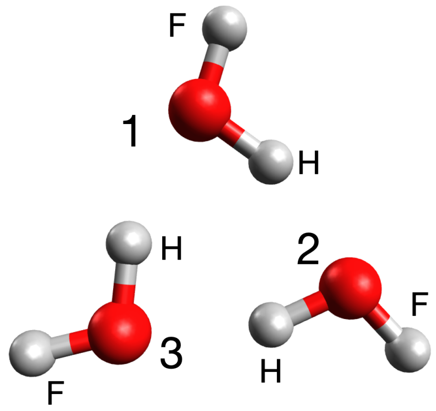

Figure S10: Minimum energy structure of the water trimer with labeling scheme used in SI tables. “B” represents a hydrogen-bonding OH bond stretch, “F” for the free OH stretch. This isomer is a cyclic planar with C_{3h} symmetry

.

Table S10: Trimer mode assignments made by visually assessing the predominant character of the optimization-based reversed mapping animations. Labeling scheme follows Figure S10. $\tilde{\nu}_{1\to 0}$ is the fundamental excitation frequency; $\Delta\langle V\rangle_{1\to 0}$ and $\Delta\langle \mathbf{T}\rangle_{1\to 0}$ are the potential and kinetic contributions, respectively. All frequencies are reported in cm^{-1}. The equivalency of the waters leads to in-phase and out-of-phase combinations of motions. Bending motions are constructed as combinations of individual monomer bending motions, with relative phases indicated by the signs. Intermolecular motions are in-plane (ip) or out-of-plane (oop) libration motions, or wagging of the free OH.

Mode	$\tilde{\nu}_{1\to 0}$	$\Delta\langle V\rangle_{1\to 0}$	$\Delta\langle T\rangle_{1\to 0}$	χ_l	Water ID number			in-phase / out-of-phase
					1	2	3	
					**OH stretch**			
20	3662	1842	1820	0.68	Asym	Asym		
19	3654	1824	1830	0.69	Asym	Asym	Asym	
18	3651	1827	1824	0.74	Asym	F	Asym	
17	3604	1804	1800	0.84	F	Sym	Sym	out
16	3591	1802	1789	0.81	Sym	B	Sym	out
15	3575	1797	1778	0.82	Sym	Sym	Sym	in
					**Bends**			
13	1474	729	745	0.70	+	−		out
12	1451	736	715	0.77	+	−	−	out
					**Intermolecular**			
11	831	495	336	0.86			oop	local
10	577	326	251	0.76	oop	oop	oop	out
9	541	261	280	0.87	oop		oop	in
8	474	293	181	0.79	ip	ip	ip	out
7	355	176	179	0.89		ip	ip	out
6	335	164	171	0.93	ip		ip	out
4	234	135	99	0.79			wag	local
3	211	162	49	0.75			wag	in
1	184	132	52	0.83	ring distortion			
0	174	113	61	0.80	ring distortion			

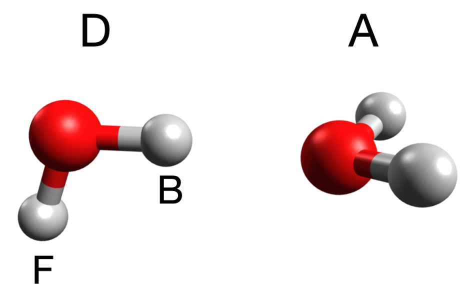

Figure S11: Minimum energy structure of the water dimer with labeling scheme used in SI tables. "B" represents a hydrogen-bonding OH bond stretch, "F" for the free OH stretch. "A" and "D" represent acceptor and donor monomer, respectively.

Table S11: Dimer mode assignments made by visually assessing the predominant character of the optimization-based reversed mapping animations. Labeling scheme follows Figure S11. $\tilde{\nu}_{1\to0}$ is the fundamental excitation frequency; $\Delta\langle V\rangle_{1\to0}$ and $\Delta\langle \mathbf{T}\rangle_{1\to0}$ are the potential and kinetic contributions, respectively. All frequencies are reported in cm^{-1}. Bending motions are constructed as combinations of individual monomer bending motions, with relative phases indicated by the signs. Intermolecular motions are in-plane (ip) or out-of-plane (oop) libration motions where the plane is defined as the donor water's plane of reflection.

Mode	Frequency	$\Delta\langle V\rangle_{1\to0}$	$\Delta\langle T\rangle_{1\to0}$	χ_l	Water ID number		in-phase / out-of-phase
					D	A	
			**OH stretch**				
11	3676	1818	1858	0.88	Asym	Asym	out
10	3646	1814	1832	0.92	Asym	Asym	out
9	3608	1824	1784	0.79	B	Sym	out
8	3577	1774	1803	0.94	Sym	Sym	in
			**Bends**				
7	1423	709	714	0.91	−1	1	out
6	1416	708	708	0.75	1	1	in
			**Intermolecular**				
5	654	237	417	0.97	oop		local
4	314	230	84	0.71	ip	ip	in
3	217	135	82	0.82	ip	ip	out
1	158	82	76	0.94		oop	local
0	59	-9	69	0.97	wag		local

Table S12: Values of $\mathrm{CIF_{intra}}$, $\mathrm{CIF_{bond}}$, and $\mathrm{CIF_{angle}}$ evaluated using the standard SVD method for different water clusters under two redundant bases.

Cluster	Redundant basis	$\mathrm{CIF_{intra}}$	$\mathrm{CIF_{bond}}$	$\mathrm{CIF_{angle}}$
Dimer	$\vec{r}_{N^2}$	−0.14	−0.17	−0.08
	$\vec{r}_{N^3}$	−0.39	−0.37	−0.42
Trimer	$\vec{r}_{N^2}$	−0.29	−0.25	−0.37
	$\vec{r}_{N^3}$	−0.65	−0.61	−0.72
Tetramer	$\vec{r}_{N^2}$	−0.53	−0.51	−0.57
	$\vec{r}_{N^3}$	−0.74	−0.71	−0.81
Hexamer	$\vec{r}_{N^2}$	−0.72	−0.70	−0.77
	$\vec{r}_{N^3}$	−0.83	−0.80	−0.89

Table S13: Values of $f_{\mathrm{inter}\to\mathrm{intra}}$ evaluated using the chemically-informed SVD method for different water clusters under two redundant bases.

Cluster	Redundant basis	$f_{\mathrm{inter}\to\mathrm{intra}}$
Dimer	$\vec{r}_{N^2}$	0.16
	$\vec{r}_{N^3}$	0.13
Trimer	$\vec{r}_{N^2}$	0.38
	$\vec{r}_{N^3}$	0.25
Tetramer	$\vec{r}_{N^2}$	0.43
	$\vec{r}_{N^3}$	0.33
Hexamer	$\vec{r}_{N^2}$	0.35
	$\vec{r}_{N^3}$	0.29

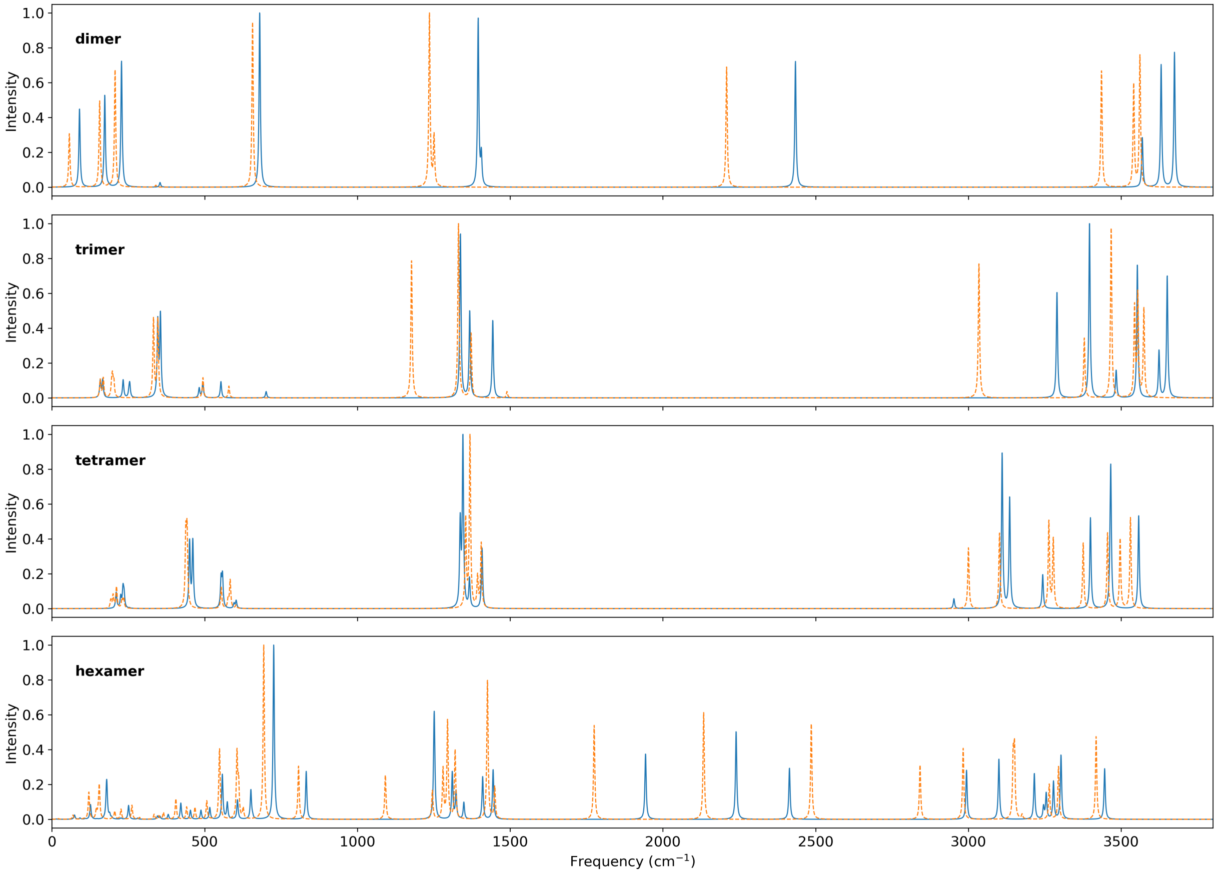

Figure S12: Predicted vibrational spectra obtained from standard SVD-based coordinate selection for the water dimer, trimer, tetramer, and hexamer, generated from $\vec{r}_{N^2}$ (solid blue), and $\vec{r}_{N^3}$ (dashed orange).

References

(1) McCoy, A. B.; Diken, E. G.; Johnson, M. A. Generating spectra from ground-state wave functions: Unraveling anharmonic effects in the OH·H2O vibrational predissociation spectrum. *The Journal of Physical Chemistry A* **2009**, *113*, 7346–7352.

(2) Mallory, J. D.; Brown, S. E.; Mandelshtam, V. A. Assessing the Performance of the Diffusion Monte Carlo Method As Applied to the Water Monomer, Dimer, and Hexamer. *The Journal of Physical Chemistry A* **2015**, *119*, 6504–6515.

(3) Wang, Y.; Bowman, J. M. Communication: Rigorous calculation of dissociation energies (D) of the water trimer, (H2O)3 and (D2O)3. *The Journal of Chemical Physics* **2011**, *135*, 131101.

(4) Mallory, J. D.; Mandelshtam, V. A. Diffusion Monte Carlo studies of MB-pol (H2O)2-6 and (D2O)2-6 clusters: Structures and binding energies. *The Journal of Chemical Physics* **2016**, *145*, 064308.

(5) Lee, V. G.; Vetterli, N. J.; Boyer, M. A.; McCoy, A. B. Diffusion Monte Carlo Studies on the Detection of Structural Changes in the Water Hexamer upon Isotopic Substitution. *The Journal of Physical Chemistry A* **2020**, *124*, 6903–6912.
